# Sewage and Organic Pollution Compounds in Nairobi River Urban Sediments Characterized by Fourier Transform Ion Cyclotron Resonance Mass Spectrometry (FT–ICR–MS)

**DOI:** 10.1021/jasms.4c00229

**Published:** 2024-09-03

**Authors:** Rory P. Downham, Christopher H. Vane, Benedict Gannon, Lydia A. Olaka, Mark P. Barrow

**Affiliations:** † Department of Chemistry, University of Warwick, Coventry CV4 7AL, United Kingdom; ‡ British Geological Survey, Organic Geochemistry Facility, Keyworth, Nottingham, NG12 5GG, United Kingdom; § Technical University of Kenya, Department of Geoscience and Environment, P.O. Box 52428-00200, Nairobi, Kenya

## Abstract

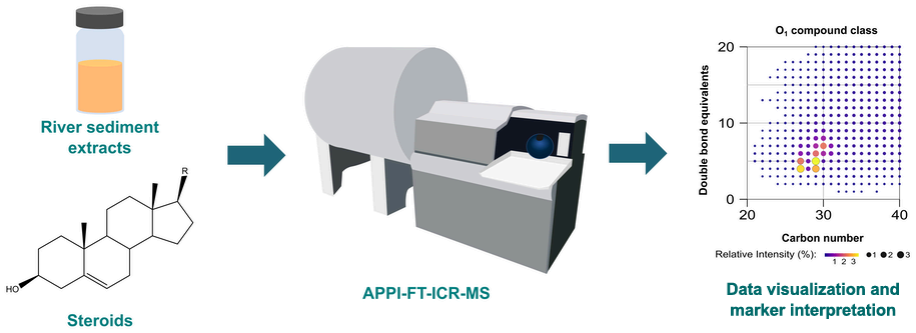

Nairobi River sediments from locations adjacent to the Kawangware and Kiambio slums were analyzed via Fourier transform ion cyclotron resonance mass spectrometry with atmospheric pressure photoionization (APPI–FT–ICR–MS). The data from these ultrahigh resolution, untargeted measurements provided new insights into the impacts of local anthropogenic activity, which included likely benzo- and dibenzothiophene pollution with a suspected petrogenic origin, and prominent surfactant-like compositions. Other features in the data included highly abundant tetra-oxygenated compounds, and oxygenated nitrogen compounds with sphingolipid interpretations. Most notably, several hydrocarbon and oxygenated compound classes in the sediment data featured intensity patterns consistent with steroid molecular formulas, including those associated with sewage contamination investigatory work. In support of this interpretation, standards of cholesterol, β-sitosterol, stigmasterol, coprostanol, cholestanol, and 5α-sitostanol were analyzed via APPI, to explore steroid ionization behavior. Generally, these analytes produced radical molecular ions ([M]^•+^), and water-loss pseudo molecular ion species ([M–H_2_O]^•+^ and [M+H–H_2_O]^+^), among various other less intense contributions. The absence of pseudo molecular protonated species ([M+H]^+^) was notable for these compounds, because these are often assumed to form with APPI. The standard measurements demonstrated how steroids can create the observed intensity patterns in FT–ICR–MS data, and hence these patterns have the potential to indicate sewage contamination in the analysis of other complex environmental samples. The steroid interpretation for the Kawangware and Kiambio data was further verified by subjecting the steroid standard radical molecular ions to collision-induced dissociation and comparing the detected fragments to those for the corresponding isolated ions from a Kawangware sediment sample.

## 1 Introduction

Nairobi, the capital city of Kenya, has experienced rapid population growth without the necessary development of facilities, services, and housing, resulting in slum dwelling proliferation.^1^ Nairobi currently has between 100 and 150 slum settlements, which are home to ca. 50–70% of the urban population.^1,2^ The Kawangware and Kiambio slums, the focus of the main study herein, are home to approximately 130 000 and 40 000 people, respectively.^2^ African cities are projected to have unprecedented annual growth rates by 2025, which will likely lead to increasing urban poverty.^3^

The definition of a slum broadly encompasses informal residential areas where households lack access to safe water, adequate living space, improved sanitation, and/or tenure security.^4^ Nairobi’s slums comprise dwellings with one or two stories, and many households are confined to a living space of ca. 9 m^2^.^1^ Dwellings are densely arranged, imparting transport and accessibility limitations that generally constrain residents to their local environment.^4^

Slum households depend upon charcoal, wood, or kerosene for cooking,^1,5^ and the latter has grown in domestic importance in Kenya due to the rising costs of wood and charcoal.^6^ The burning of these fuels within homes exposes slum residents to indoor air pollution, which can cause respiratory and pulmonary health issues.^5−7^ Outside of the home, roadways are estimated to be the dominant source of urban air pollution in developing countries.^8^ Dependence on older vehicles, less frequent vehicle maintenance, and poorer fuel quality exacerbate the challenge, with respect to more developed countries.^8^ While the local air pollution in residential areas is influenced by proximity to major roads, other human activities have been suggested to be a larger air pollution factor in slums than traffic.^9^

Slums are also often situated close to other primary pollution sources, including industrial land, and dumpsites for domestic, commercial, and industrial wastes.^1,5,10^ During the rainy season, dumpsite refuse is washed into storm sewers and transported into Nairobi’s rivers, along with redistributed pollutants from other sources.^10^ Surface water contamination investigations by Ngecu et al. in 1998 demonstrated an increase in heavy metal contamination in the Mathare, Nairobi, Ngong, and Rui Rwaka rivers after passing through industrial sites and slums.^10^

Studies concerning sediment pollution in Nairobi’s rivers have generally focused on specific contaminants. For instance, in 2018, Ndunda et al. screened for 17 organochlorine pesticides in water and sediment samples from Nairobi River, showing concentrations of some pesticides above sediment quality guidelines (SQGs) for two of the three sites investigated.^11^ Chirikona et al. recently used liquid chromatography–mass spectrometry (LC–MS) to detect 28 per- and polyfluoroalkyl substances (PFAS) in Nairobi River sediments, ascribing elevated concentrations to cottage industries situated close to the river.^12^ In 2022, Vane et al. conducted a broader river sediment pollution investigation relating to five of Nairobi’s slums; Kibera, Mathare, Kiambio, Kawangware, and Mukuru.^2^ Among other measurements, Vane et al. determined sediment concentrations of polycyclic aromatic hydrocarbons (PAHs), polychlorinated biphenyls (PCBs), dichlorodiphenyltrichloroethane (DDT), pharmaceuticals, hormones, and trace metals.^2^ The PAH ratios were consistent with vehicle exhaust, domestic kerosene, and oil-fired power generation sources.^2^ Each organic contaminant group was found generally to be between the upper and lower SQG thresholds, and of the heavy metals, only lead (Pb) concentrations were found to exceed them.^2^

Vane et al. further conducted targeted gas chromatography–mass spectrometry (GC–MS) analyses for fecal sterols and stanols (16 compounds), including coprostanol, epicoprostanol, and cholesterol, to explore anecdotal accounts of human fecal waste disposal via Nairobi’s urban rivers.^2^ River sediments from Kawangware and Kiambio were among the slums demonstrated to exhibit appreciable contributions from untreated human faeces.^2^ Slum residents are hence at risk of water borne and sediment/soil hosted diseases such as diarrhea, cholera, and typhoid.^2^ In 2017, Bauza et al. showed that the ingestion of soil was a significant transmission route for diarrhea, a leading cause of child mortality, in Nairobi’s Kibera slum.^13^

Fecal steroids, most notably coprostanol, have been studied and used as markers for fecal pollution for many decades.^14−17^ Coprostanol is a 5β-stanol, and is produced via the biohydrogenation of cholesterol by microflora in the gastrointestinal tract of mammals.^18−20^ Other 5β-stanols, similarly produced in the gut by higher mammals, are prominent biomarkers in the feces of herbivores, including those derived from the dietary plant sterols, β-sitosterol, and stigmasterol.^19−21^ 5β-Stanols must be distinguished from the 5α-stanol isomers which may be generated in the environment from the microbially mediated degradation of sterols.^18,22^ Ratios of different steroid concentrations have been explored and deployed as fecal source indicators for humans and various animals.^14,23−27^ However, as highlighted recently by Larson et al., fecal steroid ratios are not without their shortcomings, for example, due to regional and seasonal differences in diets.^23^

Steroid analysis in complex mixtures is usually targeted and often involves GC–MS or high-performance liquid chromatography–mass spectrometry (HPLC–MS).^2,18,28−30^ With GC–MS, analytes are usually first derivatized, and electron ionization (EI) is employed to produce radical molecular cations ([M]^•+^) and fragment ions.^29^ This provides structural information and enables isomeric distinction.^29^ HPLC–MS approaches typically rely on electrospray ionization (ESI), which also requires analyte derivatization because nonpolar steroids otherwise ionize poorly.^28,29^ HPLC–MS work streams use collision-induced dissociation (CID) to fragment the even-electron analyte ions, which yields limited structural information.^29^ In 2021, West and Reid reported a nano-ESI followed by ultraviolet photodissociation (UVPD) tandem mass spectrometry (MS/MS) approach, to generate sterol [M]^•+^ ions for subsequent CID analysis.^29^ The CID fragmentation spectra of the [M]^•+^ ions provided structural information similar to traditional EI fragmentation.^29^ More recently, Mueller et al. demonstrated the utility of CID for [M]^•+^ ions produced using atmospheric pressure photoionization (APPI), for a range of low molecular weight analytes (including a sterol).^31^ Mueller et al. observed ca. 65% of the number of fragments observable using EI mass spectrometry.^31^

APPI is established for steroid analysis, with several publications centered on improved analytical platforms for LC–MS.^32−38^ Steroids have been reported to form various molecular and pseudomolecular ions via APPI in positive mode, including [M]^•+^,^33,39^ protonated species ([M+H]^+^),^35,40^ odd-electron water-loss species ([M–H_2_O]^•+^),^39^ and even-electron water-loss species ([M+H–H_2_O]^+^).^32,33,36,38−40^ There is some lack of consensus here, however, differences in the compounds studied, the instrumentation, sample solvents, and data analysis all likely influence what has been observed and reported.

In this work, the same Nairobi River sediment samples characterized and analyzed previously by Vane et al.,^2^ for Kiambio and Kawangware pre-, mid-, and post-settlement locations, were subjected to analysis by APPI coupled with Fourier transform ion cyclotron resonance mass spectrometry (FT–ICR–MS). The ultrahigh resolving power and mass accuracy capabilities of this instrumentation enabled untargeted measurements, leading to detailed molecular characterizations of the complex sediments. Subsequent data visualization provided further sediment pollution insights, including likely contributions from benzo- and dibenzothiophenes and surfactant compounds. Furthermore, dominant patterns in the hydrocarbon and mono-oxygenated compound classes were observed for these sediments, which were hypothesized to pertain to steroids and related triterpenoids. A range of steroid standards were subsequently analyzed via APPI–FT–ICR–MS to confirm their ionization behavior, and support the interpretation of the sediment data. The steroid standards and corresponding suspect ions in an exemplary sediment sample were further subjected to CID, with comparisons made between the fragmentation patterns.

## 2 Experimental Section

### 2.1 Sample Details and Preparation

#### 2.1.1 Nairobi River Sediments

**Figure 1:**
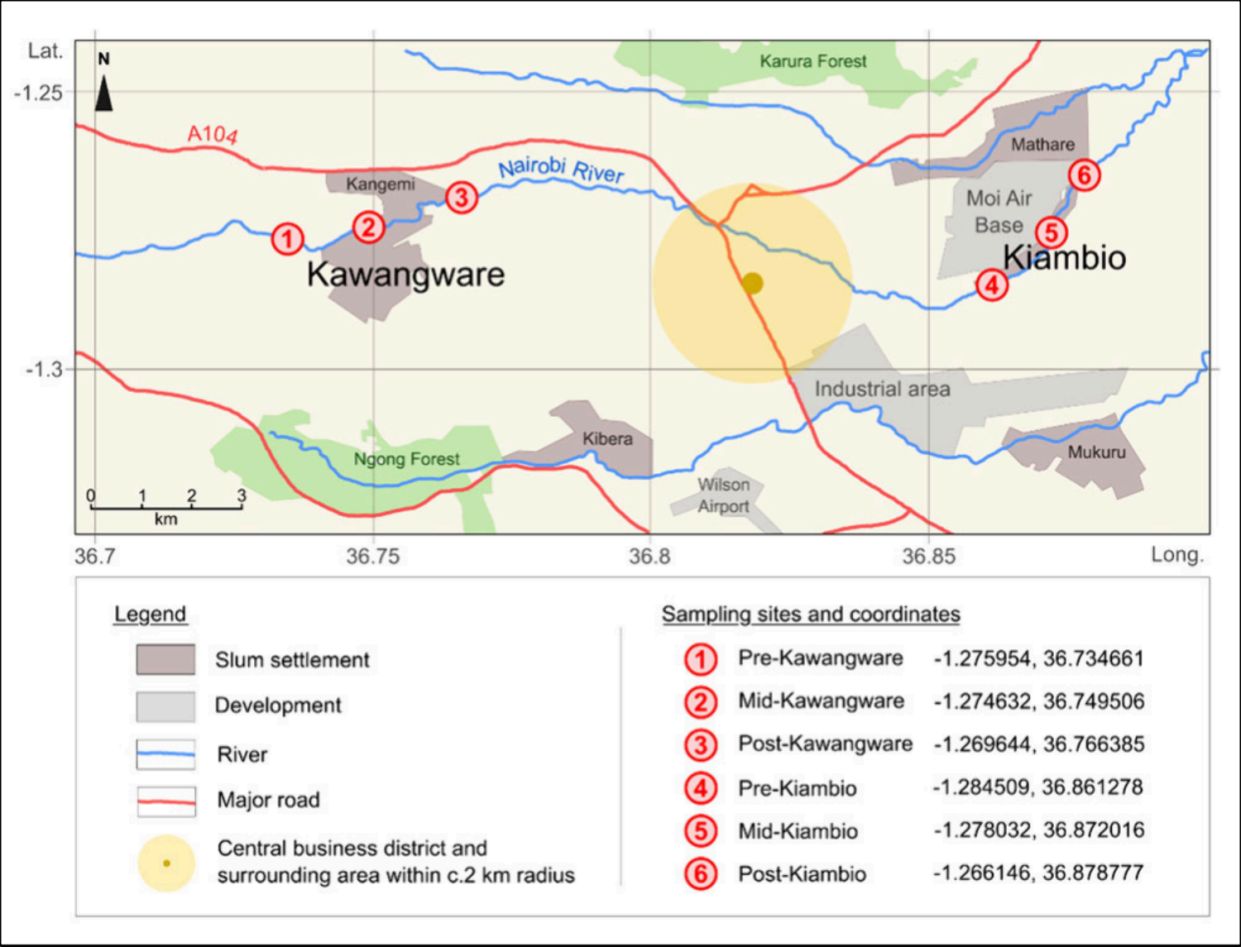
Sketch map showing the study location in Nairobi, and the sediment sampling sites. Adapted from Vane et al.^2^ Copyright 2022 Elsevier. Adapted from Bird et al.^4^ Copyright 2017 Oxford University Press.

Surface sediment samples were retrieved from the main Nairobi River channel in six locations in January 2020. These were at pre-, mid-, and post-slum sites for both Kawangware and Kiambio (Figure 1). At each location, a composite sample was generated from five subsamples collected from the corners and center of a 5 m by 5 m grid with a trowel, at 0–20 cm depth. The six composite sediment samples were sealed in polyethylene bags, transported to the laboratory at 4 °C, and then frozen at −18 °C. They were subsequently freeze-dried and sieved, with the <2 mm fraction further ground mill to <200 μm using a ball mill.

The processed sediment samples were individually extracted in dichloromethane (Fisher Scientific, Hemel Hempstead, Hertfordshire, U.K.) via accelerated solvent extraction (ASE, Dionex-300). Dichloromethane was considered appropriate for untargeted analysis given that it readily dissolves a wide range of organic compounds. For extraction, 2 g of a given sample was first mixed with copper powder and sodium sulfate in a 34 mL extraction cell. Then, after a 5 min heat-up step, a 10 min static extraction was performed at 100 °C and 1500 psi. The sediment extracts were reduced to c.5 mL under nitrogen gas, filtered individually using Whatman GF/F glass microfiber filters (GE Healthcare, Lifesciences, Pittsburgh, U.S.A.), and stored in glass vials at 4 °C. For analysis via direct infusion APPI–FT–ICR-MS, 50 μL of each extracted sediment sample was diluted with 200 μL of propan-2-ol (HPLC grade, Sigma-Aldrich, Haverhill, Suffolk, U.K.) and 200 μL toluene (HPLC grade, Honeywell, Basingstoke, Berkshire, U.K.). The propan-2-ol and toluene solvent combination is established in APPI-MS, with the protic nature of the alcohol facilitating the formation of protonated species, and toluene critically acting as a dopant in the process.^41^

#### 2.1.2 Steroid Standard Reference Materials

Cholesterol (99.7% pure, CRS, European Pharmacopoeia) and the plant compounds β-sitosterol and stigmasterol (>99% pure, Avanti Polar Lipids, Alabaster, AL, U.S.A.) were selected as exemplar sterols for analysis. Coprostanol (≥98% pure, Sigma-Aldrich, Haverhill, Suffolk, U.K.), cholestanol and 5α-sitostanol (>99% pure, Avanti Polar Lipids, Alabaster, AL, U.S.A.) were also purchased as representative stanols (see Table S.1 for more details, Supporting Information, SI). These steroids were individually prepared for FT–ICR–MS analysis as 0.01 mg/mL solutions in 1:1 (v/v) propan-2-ol and toluene (HPLC grade, Honeywell, Basingstoke, Berkshire, U.K.). An additional cholesterol sample was also prepared as a 0.01 mg/mL solution in 2:2:1 (v/v/v) propan-2-ol, toluene, and dichloromethane (unstabilized HPLC grade, Fisher Scientific, Hemel Hempstead, Hertfordshire, U.K.), to investigate whether dichloromethane influenced ionization behavior. This was undertaken because of the environmental and safety considerations surrounding the use of dichloromethane.

### 2.2 FT–ICR–MS

#### 2.2.1 Sediment Samples via Broadband

The six sediment samples were analyzed using a 12 T solariX FT–ICR mass spectrometer (Bruker Daltonik GmbH, Bremen, Germany), following external instrumental calibration with a solution of sodium trifluoroacetate (98%, Sigma-Aldrich, Haverhill, Suffolk, U.K.) in 50:50% v/v toluene and methanol (HPLC grade, Honeywell, Basingstoke, Berkshire, U.K.). The measurements were performed in positive-ion mode via an APPI II source (Bruker Daltonik GmbH, Bremen, Germany), on the same day. Solvent blanks (a mixture of dichloromethane, propan-2-ol, and toluene) were analyzed between samples to monitor for carry over, and a sediment sample doped with sodium trifluoroacetate was also measured for internal calibration purposes.

The samples were directly infused from a 1 mL syringe with a flow rate of 600 μL/h. The temperature of the source vaporizer was maintained at 350 °C, the nitrogen nebulizing gas pressure was 1.3 bar, and the nitrogen drying gas was set to 4.0 L/min and 230 °C. Ionization was initiated by the APPI source krypton lamp, emitting photons at 10.0 and 10.6 eV, and the generated ions were drawn into the transfer capillary, held at −2000 V. The capillary exit was set to 220 V, and the deflector was at 200 V. Funnel 1 was set to 150 V, funnel 2 was set to 6.0 V, and skimmers 1 and 2 were held at 10.0 and 5.0 V, respectively. The funnel RF amplitude was 100 V_pp_, and the quadrupole was used in radio frequency only mode. Ions were accumulated in a hexapole collision cell for 0.06–0.2 s, depending on the sample, before being delivered to an infinity cell analyzer via a transfer hexapole, operated at 4 MHz and with a 1.0 ms time-of-flight. The front and rear trapping plates of the infinity cell were set to ca. 0.4 V, and ions were detected over the range *m*/*z* 147.4–3000. Each acquired mass spectrum represents 260–300 summed scans of 4 million data points, subsequently zero-filled and apodized (full sine), and converted from the time domain by the Fourier transform. The resolving power achieved was approximately 680 000 FWHM, at *m*/*z* 300 (magnitude mode).

#### 2.2.2 Steroid Standard Samples via Broadband

The sterol and stanol standard samples were analyzed using the 12 T solariX FT–ICR mass spectrometer with parameters similar to those given in Section 2.2.1 (including the vaporizer temperature at 350 °C, which was considered to be typical). The instrument was once again externally calibrated using sodium trifluoroacetate, and the acquired standard data sets comprised 100–200 summed scans.

Further analyses were performed with the cholesterol standard to explore ionization behavior across a range of APPI source vaporizer temperatures, with *T* = 200 °C, 250 °C, 300 °C, 350 °C, and 400 °C. The results from this investigation have been included in Figure S.1 (SI).

#### 2.2.3 CID Analysis

Isolation spectra for the steroid sample [M]^•+^ ions were acquired using the 12 T solariX FT–ICR mass spectrometer, following the broadband analyses (see Section 2.2.2). This was achieved by setting the quadrupole isolation window to 1.5 Da, and the Q1 mass to *m*/*z* 386.35 for cholesterol, *m*/*z* 414.39 for β-sitosterol, *m*/*z* 412.37 for stigmasterol, *m*/*z* 388.37 for coprostanol and cholestanol, and *m*/*z* 416.40 for 5α-sitostanol. The ion accumulation times were increased to 0.3–1.0 s, and 50–150 scans were acquired per spectrum, as appropriate. Ions in the mid-slum Kawangware sample with *m*/*z* corresponding to the six steroid standard [M]^•+^ ions were also isolated using a window size of 1.5 Da, and the same Q1 mass set points as for the standards. An ion accumulation time of 0.6 s was set for all peaks, and isolation spectra were acquired with 50–60 scans.

After each steroid standard compound and Kawangware isolation spectra had been acquired, the 12 T solariX FT–ICR mass spectrometer was further tuned to improve the transfer and detection of lower *m*/*z* fragment ions. This included increasing the transfer hexapole frequency to 6 MHz, decreasing the ion flight time to 0.5 ms, and adjusting the low *m*/*z* detection to 92.13. With the described quadrupole isolation parameters in effect, CID was performed with collision voltages between −13 and −20 V for the sterol standards, and −8 V for the stanol standards (reflecting optimum values from a tested range). The collision voltages used for the Kawangware ion isolations were guided by those used for the standard compounds with corresponding *m*/*z*. CID spectra for the standard compounds were acquired over 100–130 scans (with 300 scans for cholesterol), and 100 scans were acquired for the Kawangware CID spectra.

### 2.3 Data Processing and Analysis

The broadband sediment data sets were phased using FTMS processing v2.2.0 (Bruker Daltonik GmbH, Bremen, Germany) to obtain absorption mode mass spectra. Kilgour apodization was applied, using Kilgour max values in the range of 0.11–0.27, followed by baseline correction. Phasing was not employed for the steroid broadband spectra, or CID spectra, as these were far less complex.

The sodium trifluoroacetate-doped sediment mass spectrum was recalibrated against the dopant peaks, using DataAnalysis 5.0 (Bruker Daltonik GmbH, Bremen, Germany). A calibration list was subsequently created from a protonated mono-oxygen (O_1_[H]) and protonated hydrocarbon (HC[H]) class homologous series (Table S.2, SI) for internally recalibrating all broadband sediment spectra in DataAnalysis 5.0. Recalibrated spectra were then exported for processing in Composer 1.5.6 (Sierra Analytics, Modesto, CA, U.S.A.), where sample peaks were assigned molecular formulas within a matching tolerance of 1 ppm by searching for homologous series (with CH_2_ building units) using the elemental constraints: C = 0–200, H = 0–1000, O = 0–12, S = 0–2, N = 0–2, P = 0–1, Cl = 0–2. These ranges were deployed in a CHO > CHOS > CHON > CHOSNPCl priority sequence (after extensive testing in other configurations). For each sediment sample, the assigned molecular formulas were categorized by compound class, shown in terms of the number of heteroatoms, e.g., “O_1_” accounts for all organic compounds with one oxygen atom (“HC” represents heteroatom-free hydrocarbon compositions). Given that APPI generates both radical ions and protonated species in positive-ion mode, with analytes having the potential to form both,^41,42^ classes with the “[H]” label denote protonated species, whereas those without the label represent radical cations. The sample peaks assigned as pure hydrocarbons or with 1–2 oxygen atoms that were also present in ASE, filter paper, and solvent blank FT–ICR mass spectra, at >20% base peak intensity (as per other work^43^), were subtracted from sediment sample mass spectra. For all other assignment classes, sample peaks also occurring in blanks at >1% base peak intensity were subtracted,^44^ unless of special interest and of greater relative intensity in at least one sample spectrum. It is acknowledged that this subtraction procedure may have eliminated a limited number of genuine sample peaks. Finalized peak assignments for the sediments were subsequently processed using KairosMS software^45^ (University of Warwick, Coventry, U.K.) for creating data visualizations.

The steroid standard broadband spectra were plotted in magnitude mode and recalibrated using DataAnalysis 5.0 using four peaks. Cholesterol was the first sample recalibrated in this way, from peaks representing [M]^•+^, [M–H_2_O]^•+^, [M+H–H_2_O]^+^, and [M+H–4H]^+^ molecular and pseudo molecular species (Table S.3 in the SI), which were reported previously by van Agthoven et al.^39^ Peaks for equivalent (or closely related) species were chosen as calibration points for the other steroid standard broadband samples (Table S.3 in the SI). Any ions in the spectra also occurring (with respectable intensity) in solvent blanks were regarded as contaminants.

The APPI isolation spectra for the steroid standard [M]^•+^ ions and Kawangware sample peaks were plotted in magnitude mode and recalibrated using a single point correction in DataAnalysis 5.0. The CID spectra were processed in the same way, but then further recalibrated to lists of fragment ions based on previously reported fragments for cholesterol^29^ and β-sitosterol,^46^ or fragments with frequently observed losses. These fragment recalibration lists (Table S.4) were also used for the Kawangware CID spectra.

Recalibrated steroid standard broadband spectra and all recalibrated CID fragmentation spectra were processed in Composer 1.5.6. Assignments were made within a 1 ppm matching tolerance, and with the elemental constraints configured for CHO compositions only. Isolated assignments were permitted and were confirmed using the “SmartFormula Manually” tool in DataAnalysis 5.0. Even-electron species assigned for broadband spectra were assumed to be protonated species.

## 3 Results and Discussion

### 3.1 Compound Class Distributions for the Sediment Samples

**Figure 2:**
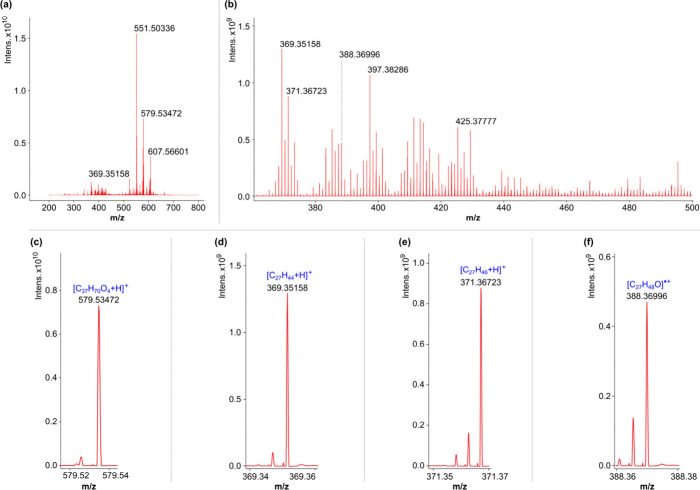
(+)APPI–FT–ICR mass spectrum (phased) for the mid-Kiambio sediment sample: (a) broad overview; (b) expanded ca. *m*/*z* 360–500 region with intense contributions from suspected steroid ions; and (c–f) example peaks from selected classes of interest. The *m*/*z* values given are post recalibration.

After phasing the data, the average resolving power achieved across all mass spectra at *m*/*z* 300 was ca. 1 100 000. This enabled ca. 3000–ca. 14 500 (mean ca. 9500) monoisotopic assignments per sediment spectrum, with root-mean square errors between 0.27–0.33 ppm. An overview of the mid-Kiambio sediment spectrum is provided in Figure 2, with exemplar peaks from selected compound classes of interest (*vide infra*) shown to higher magnification. Further mass spectrum overviews are provided in Figure S.2 (SI).

**Figure 3:**
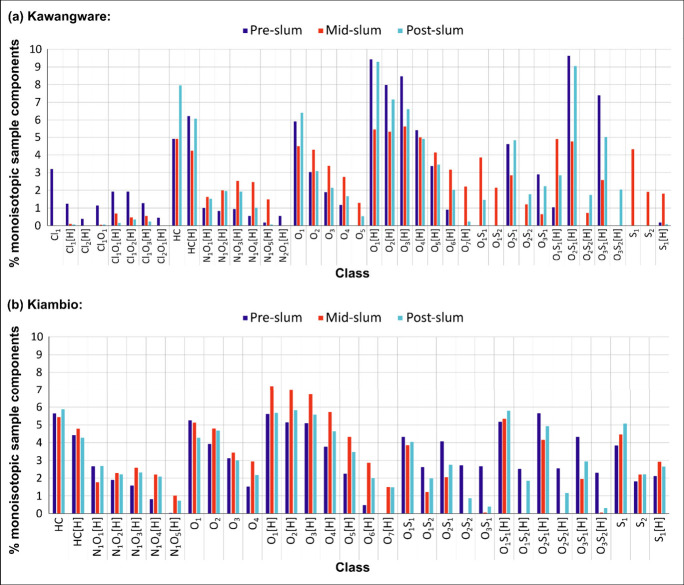
APPI–FT–ICR–MS class distributions for the (a) Kawangware sediments and (b) Kiambio sediments.

The assigned molecular formulas for each slum location are represented in the compound distribution in Figure 3, which provides the number of monoisotopic molecular components for each compound class, expressed as percentages of the overall number of monoisotopic components in each sample. Only classes with a monoisotopic signal intensity contribution of ≥0.4% in at least one slum sample were included, and for the purposes of this data representation, blank peaks with >1% relative intensity were removed from all classes.

Figure 3 shows that relatively high numbers of components in the sediments were accounted for by the HC, HC[H], O_1_[H] – O_4_[H], O_1_S_1_[H] and/or O_2_S_1_[H], and (where present) S_1_ classes. Relatively high component percentages were also associated with the O_X_ classes, but typically to a less extent than the O_X_[H] classes. The N_1_O_1_[H] – N_1_O_5_[H] classes accounted for fewer numbers of components generally, but are notable for their presence in most samples. The Kawangware plot also features an interesting distribution of organochlorine classes which, if not for the numbers observed for the pre-Kawangware location, would have been below the threshold for reporting.

The class distributions were examined for bar height patterns across the pre-, mid-, post-slum sites which might indicate the introduction of compounds at the slum (considering river flow in a pre-slum to post-slum direction). This type of relationship was apparent for the O_2_ – O_4_, O_5_[H] – O_7_[H], N_1_O_2_[H] – N_1_O_5_[H], S_1_, S_2_, S_1_[H], and O_1_S_1_[H] classes for both slums. Section 3.2 provides a deeper analysis of the compound classes with notable features and patterns.

### 3.2 Sediment Compound Classes with Noteworthy Features

#### 3.2.1 Suspected Steroid Signatures in the HC, HC[H], O_1_ – O_2_, and O_1_[H] – O_3_[H] Classes

**Figure 4:**
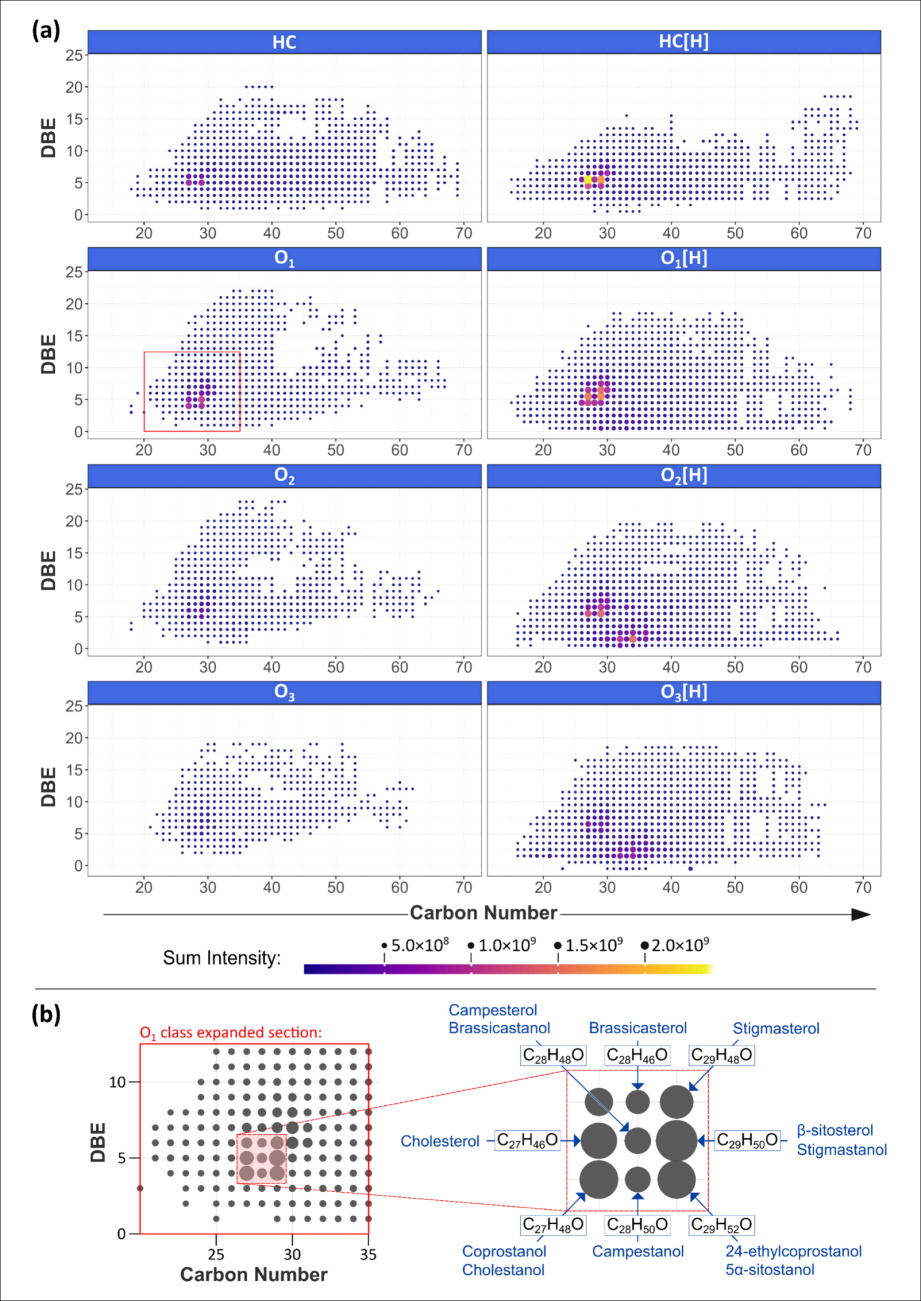
(a) DBE versus carbon number plots for compound classes (monoisotopic) with relatively intense, likely steroid compound contributions, for the mid-Kawangware data. (b) Enlarged region of the O_1_ class DBE plot showing intense data points and possible steroid assignments.

Plots of double bond equivalents (DBE) versus carbon number for the HC, HC[H], O_1_ – O_3_, and O_1_[H] – O_3_[H] classes for the mid-Kawangware data are given in Figure 4 (a). The corresponding plots for the other sediment samples were similar, albeit with some intensity variations between the more prominent data points. DBE represents the number of rings and double bonds involving carbon,^47^ thus these plots visualize chemical space within compound classes. The DBE versus carbon number plots provided in Figure 4 (a) feature regions of high intensity around DBE of ca. 4–7 (half integer values for protonated species) and carbon number ca. 27–30. The particularly intense HC[H] class C_27_ data point with DBE of 5.5 represents the [C_27_H_44_+H]^+^ species (see Figure 2 (d) for example peak), which could correspond to the analyte cholesta-3,5-diene or a related isomer. Cholesta-3,5-diene is a steradiene, which form in water and sediments from sterols,^48^ and its presence would be logical given that fecal steroid concentrations, including cholesterol, were shown to be high in most of these sediments previously by Vane et al., using GC–MS.^2^ It is also possible that [C_27_H_44_+H]^+^ was generated from cholesterol in the sample during ionization. Indeed, when analyzing a cholesterol standard via APPI–FT–ICR–MS, alongside the molecular ion [C_27_H_46_O]^•+^, van Agthoven et al. detected [C_27_H_44_+H]^+^, which was inferred to be the result of water-loss from the molecular protonated species.^39^

The O_1_ class DBE versus carbon number plot in Figure 4(a) features a relatively intense C_27_ data point with DBE of 5 (also observed for the other samples), assigned as [C_27_H_46_O]^•+^, which could be interpreted as the cholesterol molecular ion, [M]^•+^. It follows that the other intense O_1_ class data points may also correspond to steroid molecular ions, given the measurements made by Vane et al.,^2^ with [C_27_H_48_O]^•+^ (C_27_, DBE = 4), [C_29_H_50_O]^•+^ (C_29_, DBE = 5), and [C_29_H_52_O]^•+^ (C_29_, DBE = 4) potentially representing coprostanol, β-sitosterol, and 24-ethylcoprostanol, respectively. Figure 4 (b) shows an enlargement of the O_1_ class DBE versus carbon number plot pattern of intense signals, with further possible steroid isomers indicated. The relative abundance of some of the more intense O_1_ class suspected steroid signals, for all sediments, is provided in Figure S.3 (SI). The mid-Kawangware and post-Kawangware sites had more intense “cholesterol”, “coprostanol”, and “β-sitosterol” signals than pre-Kawangware, and a similar (although less pronounced) trend was similar for the set of Kiambio samples. If the peaks do represent these steroids, and relative abundance is reflective of concentration (although other parameters are influential), then this might indicate sewage input at the mid-slum sites, particularly for the case of Kawangware. It is, however, important to acknowledge that various steroids can be expected to occur naturally in soils and sediments unaffected by human sewage, for example, from vegetation.^49^

Considering the relationship between cholesterol and cholesta-3,5-diene, the HC[H] class DBE versus carbon number intensity pattern in Figure 4 could represent the water-loss derivatives of a range of steroids, either due to processes in the environment or the APPI source. The occurrence of the patterns in the O_1_[H] class is intriguing, as an obvious interpretation would be protonated molecular steroid species, however, the protonated species for cholesterol was not detected via APPI by van Agthoven et al.^39^ Furthermore, the similar intensity patterns in the O_2_, O_2_[H], and O_3_[H] classes could potentially indicate oxysterols, or other triterpenoids. To further understand these data, the ionization behavior of steroid standards via APPI was investigated (see Sections 3.3–3.5).

#### 3.2.2 O_4_[H] Class

The O_4_[H] class was dominated by the three peaks from a homologous series with DBE of 2.5, with *m*/*z* 551.50339, 579.53469, and 607.56599 (calculated values), assigned as [C_35_H_66_O_4_+H]^+^, [C_37_H_70_O_4_+H]^+^, and [C_39_H_74_O_4_+H]^+^, respectively (see Figure 2(a) and (c)). These peaks were dominant in all samples, although notably less so for pre-Kawangware (Figure S.2). The compounds that these ions were generated from are likely diacylglycerol-type lipids, although it was not possible to obtain further conclusive structural insights within the scope of this work.

#### 3.2.3 S_1_ Class

**Figure 5:**
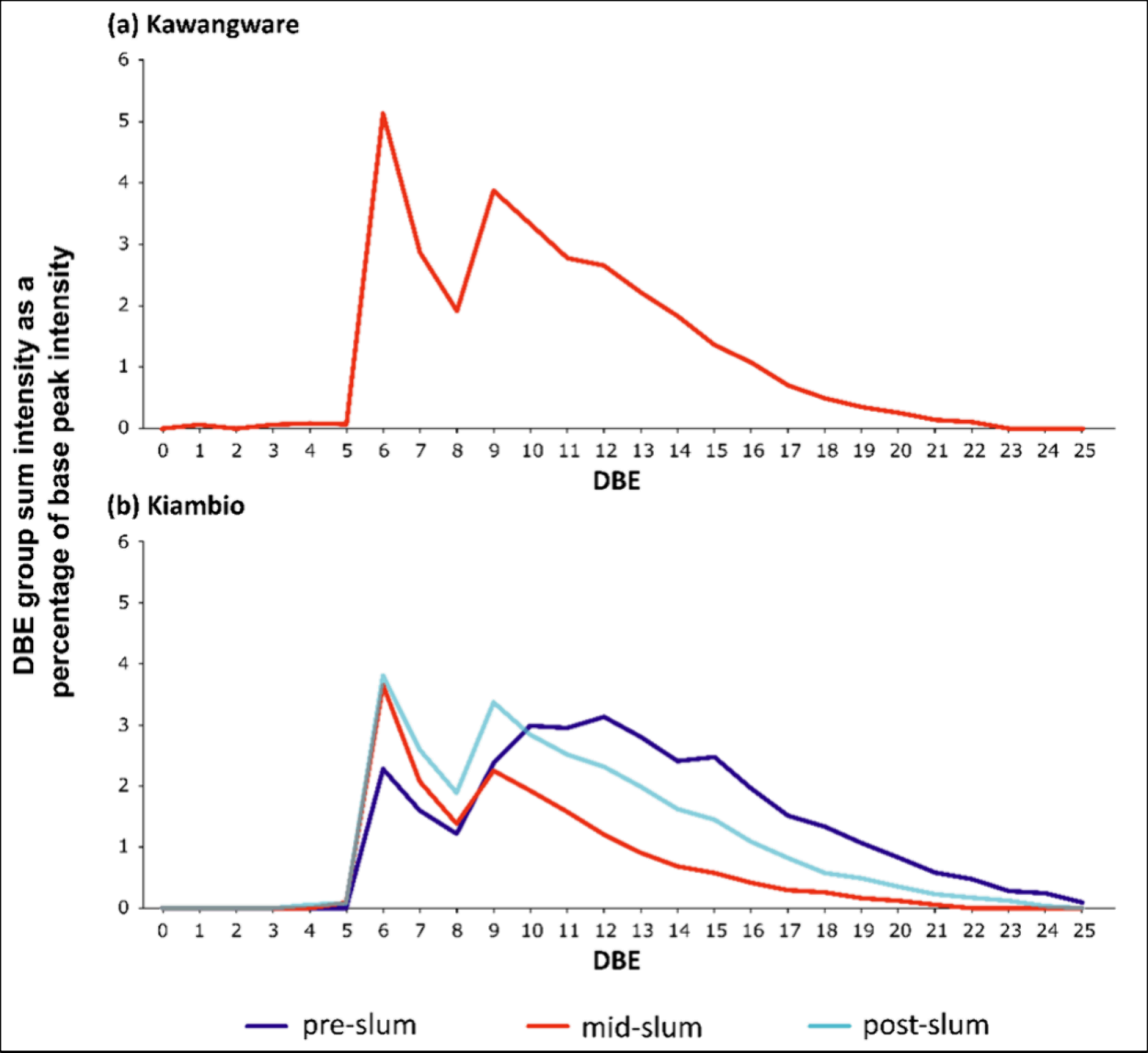
S_1_ class summed relative intensities by homologous series (monoisotopic contributions) for (a) Kawangware and (b) Kiambio.

The S_1_ class in the Nairobi sediments revealed a notable pattern for the mid-Kawangware, mid-Kiambio, and post-Kiambio sites; relatively intense homologous series with DBE of 6 and 9, see Figure 5. Such features are usually related to the thiophenic distributions observed in the S_1_ class in petroleomic data, with intense series with DBE of 6, 9, and 12.^50,51^ The spacing of DBE = 3 between predominant homologous series is indicative of fused aromatic rings, as would be seen for benzothiophenes, dibenzothiophenes, and benzonaphthothiophenes.^50^ Radović et al. recently detected dominant S_1_ class species with DBE of 6, 9, and 12 in sediments from the Pearl River estuary, China, interpreted in terms of thiophenic compounds from petrogenic sources.^52^ Similar thiophenic patterns were also observed by Thomas et al., in a sediment core from Staten Island, U.S., and were ascribed to possible contamination by petroleum-related compounds.^53^

Although the series with DBE of 6 and 9 are intense for the mid-Kwangware, mid-Kiambio, and post-Kiambio samples, the DBE = 6 series, which are generally most intense from C_26_–C_44_, are more prominent than the DBE = 9 series, typically most intense from C_29_–C_43_ (see Figure S.4, SI, for alternative data representation). Assuming that the compounds with DBE of 6 and 9 are benzothiophenes and dibenzothiophenes, their occurrence could relate to fuel combustion,^54^ however, the length of the homologous series suggests many alkyl derivatives are present, which is more reflective of a transport fuel source.^55^ Moreover, the dominance of the DBE = 6 series might be indicative of kerosene fuel contamination, since benzothiophene and its alkylated forms are suggested to be the main sulfur compounds in kerosene and related fuel oils.^56^

The pre-Kiambio site data also featured an intense S_1_ DBE = 6 series similar to the other Kiambio sites (Figure 5). The DBE = 9 series in the pre-Kiambio data is also relatively abundant, however, there is a broad rise in intensity across the series with DBE of 9–14 (Figure 5). The peak intensity for this rise is centered on DBE of 12, which could be indicative of benzonaphthothiophene contributions. This closer proximity of the pre-Kiambio site to both the central business district and the industrial zone to the south might account for these contributions at higher DBE (Figure 1).

The absence of an S_1_ class in the pre-Kawangware and post-Kawangware site data suggests that the likely thiophenic contamination was introduced at the Kawangware slum and was not transported downstream. The indiscriminate occurrence of S_1_ class contributions across all Kiambio sites might reflect the influence of the industrial area (to the north of Mukuru) or the Moi Air Base, both of which are much closer to Kiambio than Kawangware (Figure 1).

#### 3.2.4 O_2_S_1_[H] and O_3_S_1_[H] Classes

**Figure 6:**
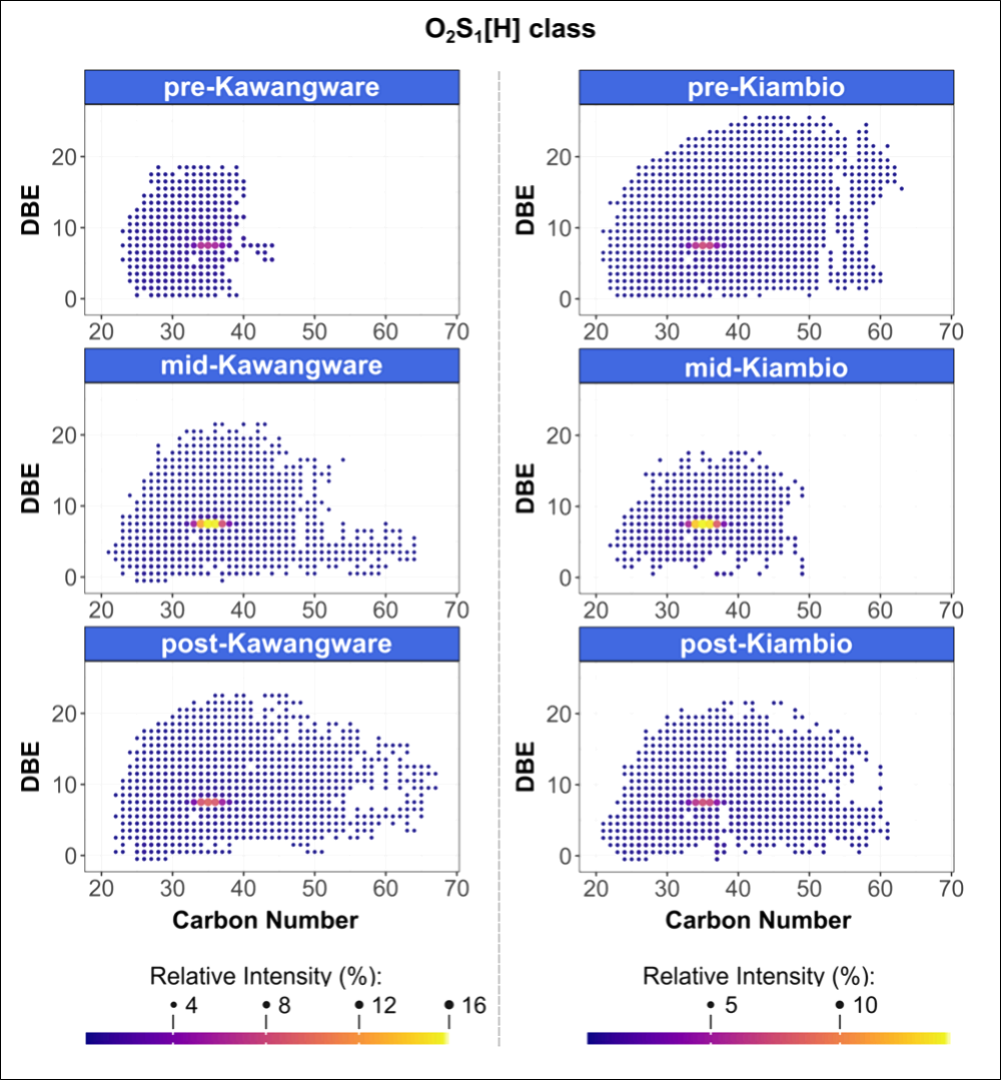
DBE versus carbon number plots for the O_2_S_1_[H] class (monoisotopic assignments).

Plots of DBE versus carbon number for the O_2_S_1_[H] classes from all sediment locations are provided in Figure 6. These class plots feature a high intensity pattern in the 7.5 DBE homologous series, between C_32_–C_38_, with highest relative abundance observed for the mid-slum sites. The most intense peak in this series represents the neutral analyte C_35_H_56_O_2_S. It is hypothesized that this compound, and the close homologues, are surfactant compounds, and alkyl phenate sulfides (or “phenates”) offer an intriguing match for the molecular formulas.^57^ These compounds are engine oil lubrication additives,^57^ and they have been associated with calcium ions in particulate matter from traffic-affected environments.^58^ The abundant O_2_S_1_[H] peak series observed in the Nairobi sediments was also previously detected in river sediments from the Pearl River estuary, China, via APPI–FT–ICR–MS, although the authors did not speculate on the origin of these signals.^52^

The O_3_S_1_[H] class also featured relatively intense homologous series, with DBE of 6.5 and 8.5, within similar carbon number ranges as for the intense O_2_S_1_[H] class series (see Figure S.5 for DBE versus carbon number plots). It is speculated that these O_3_S_1_[H] species are therefore related to the O_2_S_1_[H] species, either owing to oxidation of the O_2_S_1_ class analytes in the environment, or due to ionization pathways.

#### 3.2.5 N_1_O_2_[H] and N_1_O_3_[H] Classes

**Figure 7:**
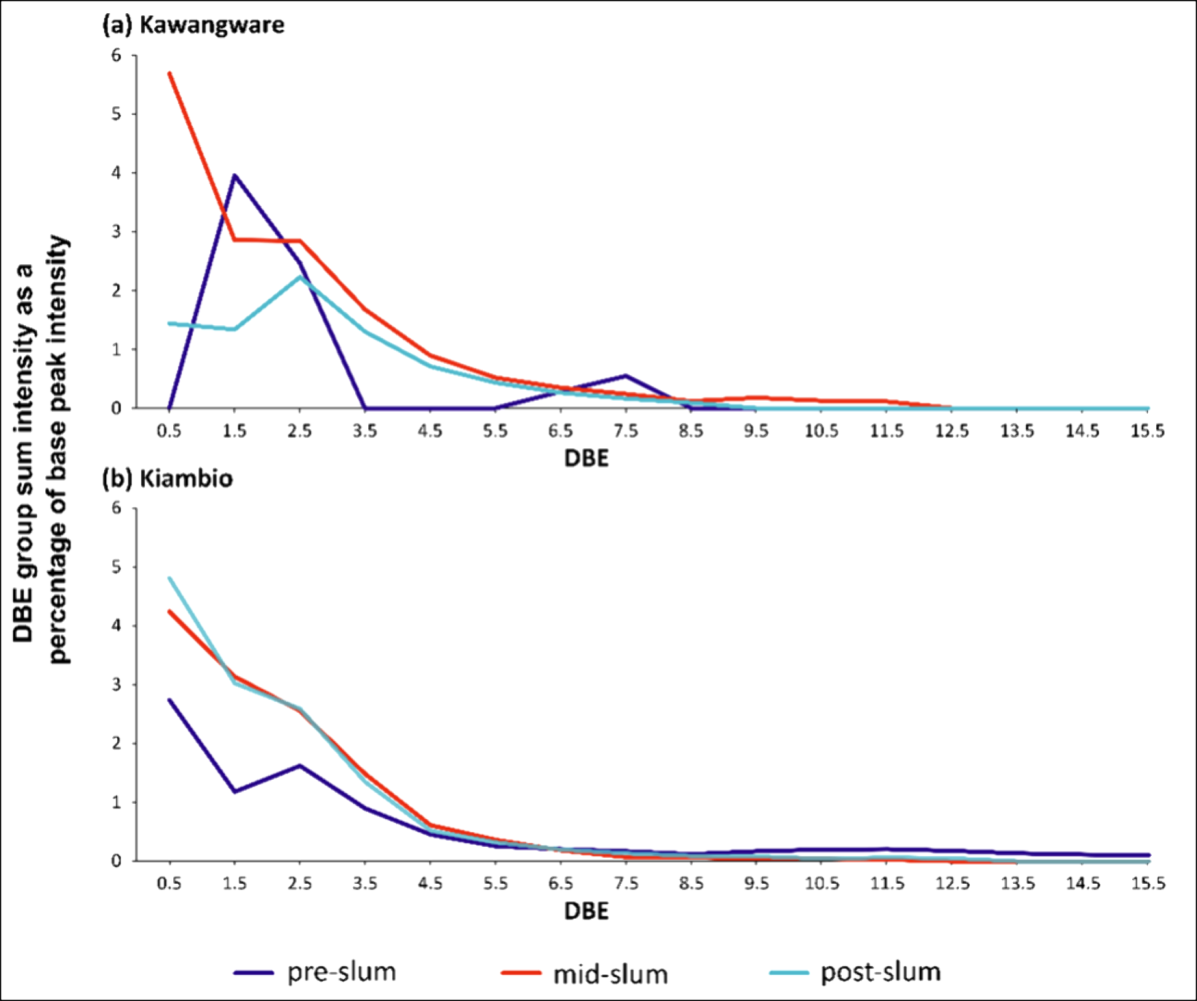
N_1_O_3_[H] class summed relative intensities by homologous series (monoisotopic contributions) for (a) Kawangware and (b) Kiambio. N.B. homologous series limited to carbon number range 30–40 only.

Within the N_1_O_2_[H] class, the more saturated DBE = 1.5 and DBE = 2.5 homologous series were relatively intense over the approximate range of C_32_–C_36_, for all sampling locations, except for pre-Kawangware where the DBE = 2.5 homologous series was not prominent (see Figure S.6 for DBE versus carbon number plots). The C_34_ compound in the DBE = 2.5 series was often of highest relative abundance for the class. The N_1_O_3_[H] class also exhibited high intensity patterns associated with the more saturated compounds, centered around the [C_35_H_71_NO_3_+H]^+^ species (DBE = 0.5) for most sample locations. Figure 7 displays the intensity patterns across the various homologous series of the N_1_O_3_[H] classes for all sediment locations, over the range of C_30_–C_40_.

Similar N_1_O_2_[H] and N_1_O_3_[H] class intensity signatures have been detected by Radović et al. in sediments from the Pearl River estuary, China.^52^ Radović et al. tentatively interpreted these contributions as sphingolipid-related, postulating that marine microorganisms could be the origin of these compounds.^52^ Sphingolipids are hence hypothesized to account for the intense contributions to the more saturated homologous series of the N_1_O_2_[H] and N_1_O_3_[H] classes observed for the Nairobi sediments. Indeed, [C_35_H_71_NO_3_+H]^+^ could represent a dihydroceramide sphingolipid, which are precursors to more complex sphingolipids.^59^ However, a marine organism source is rejected herein owing to the geographical setting of Nairobi. Sphingomonas bacteria, widely distributed naturally in water and soil, could be a respectable alternative source of sphingolipids.^60^ Dihydroceramide was demonstrated by Burenjargal et al. to play an important function in the physiology of the Chungbukensis Sphingomonas strain, which was isolated from sediments impacted by industrial pollution.^61^ Indeed, Sphingomonas are well adapted to environments contaminated with organic pollutants,^60,62^ and hence may be present in elevated numbers in the Nairobi River sediments. An alternative bacterial origin is one related to fecal contamination, because dihydroceramides are also associated with the Bacteroidetes phylum that colonize the large colon of mammals.^63,64^ It is estimated that the colonic microbiome contains 1 g of sphingolipids derived from bacteria at any time.^63^ The abundance of sphingolipids in sewage treatment sedimentation ponds has been demonstrated,^65^ and recently Zhu et al. considered the extraction of ceramides from sewage sludge.^66^ Therefore, the N_1_O_2_[H] and N_1_O_3_[H] marker patterns in the Nairobi sediment DBE versus carbon number plots could be related to sphingolipids, introduced into the river via human sewage.

### 3.3 Ionization Behavior of Steroid Standards via APPI

To further understand the prominent, suspected steroid features in the HC, HC[H], O_1_–O_2_, and O_1_[H]–O_3_[H] class data (Section 3.2.1), the ionization behavior of a selection of steroids was investigated. APPI overview spectra for the six steroid standards have been provided in Figures S.7–S.12 (SI). The ion species detected via broadband mode (directly infused in propan-2-ol and toluene) have also been tabulated and included in the SI (Tables S.5–S.10). Concerning the comparison between the solvent system with and without dichloromethane for cholesterol, the same ions listed in Table S.5 were also detected for the sample with dichloromethane, and no further ions with relative abundance >1% were observed.

**Figure 8:**
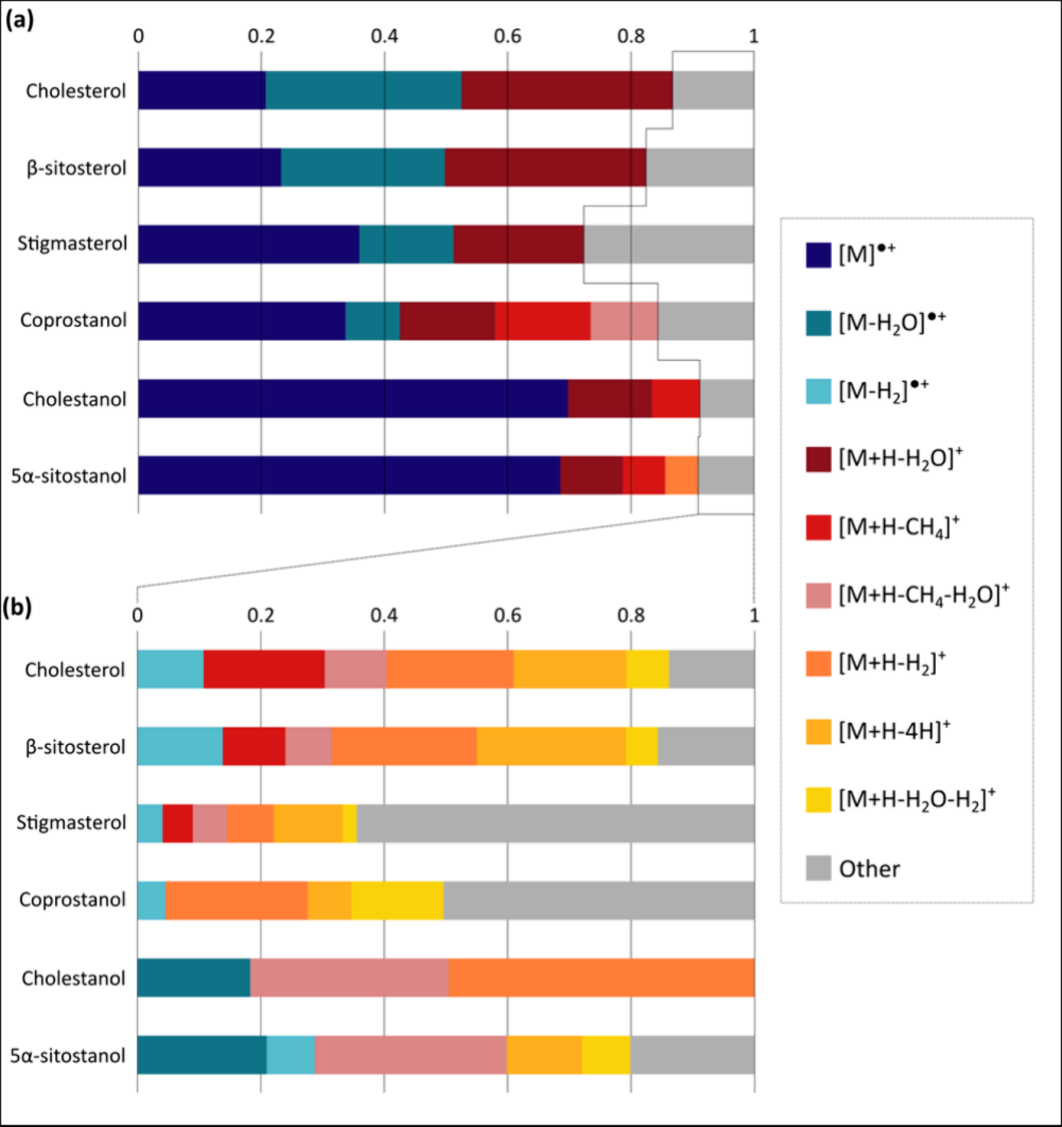
Scaled signal contributions for ion species detected for individual steroids via broadband APPI. The major ion species (accounting for >5% overall signal) are shown in (a), and minor ion species are provided in (b).

Figure 8 provides a visual summary of the ion species produced for the standard compounds. For each steroid, the intensities of all peaks (monoisotopic) with >1% relative abundance were summed (excluding contaminants and unknowns), and the intensities of each of these ions were divided by the summed totals. Ions with a subsequent signal intensity contribution >5% were classified here as “major” and incorporated into Figure 8 (a), whereas the more common “minor” ion species are given in Figure 8 (b). Generally, the major ions–radical molecular ions and two water-loss molecular ion species–were similar in relative contributions for the sterols. The major ion species were also very similar in terms of relative signal contributions between cholestanol and 5α-sitostanol, which favored molecular radical ion formation, whereas coprostanol formed a greater variety of major ion species. Interestingly, ions interpreted as methane-loss protonated species were detected with greater relative signal intensities for the stanols than for the sterols.

Oxidized species were included among the minor ions detected for cholesterol, β-sitosterol, and coprostanol. [M+H+O–H_2_]^+^ was detected for cholesterol, [M+H+O–H_2_]^+^ and [M+H+O_2_]^+^ were observed for coprostanol, and [M+O–H_2_]^•+^ was detected for β-sitosterol. Oxidized species may have also been measured for the other steroid standards, but below 1% abundance. These results suggest that steroid oxidation via APPI is relatively limited (for these standards at least), and hence more intense oxysteroid peaks in complex mixture mass spectra may represent sample analytes. Finally, it is interesting to note that molecular protonated species ([M+H]^+^) were not detected for any of the steroid standards via APPI. It is possible that molecular protonated species were formed but fragmented in the ion source region to produce the various even-electron ion species detected.

### 3.4 Significance of Steroid APPI Behavior for Complex Mixture Analysis

The sterol and stanol standards analyzed in these studies were shown to form molecular radical ions ([M]^•+^) via APPI, as a major ionization mode. Therefore, examining the O_1_ class is an appropriate first step when considering the significance of steroid contributions in complex sediment data. As discussed, the intensity pattern noted in the O_1_ class DBE versus carbon number plot also occurred in the HC and HC[H] classes (Figure 4). It was demonstrated that this is consistent with steroid ionization, because both [M–H_2_O]^•+^ and [M+H–H_2_O]^+^ are favored ion species formed via APPI (although [M–H_2_O]^•+^ was far less prominent for cholestanol and 5α-sitostanol). The occurrence of a similar intensity pattern in the O_1_[H] class (Figure 4) is, however, more difficult to account for, as it implies the formation of [M+H]^+^ species which were not detected for the standard steroids analyzed herein. These intense data points could instead be explained in terms of the CH_4_-, H_2_-, and 4H-loss protonated species from other steroids, as detected for the standards in this study. It is also possible that the O_1_[H] class represents oxidized steroid protonated species which lost H_2_O before detection. The occurrence of other CH_4_- and H_2_-loss protonated species with additional H_2_O losses further demonstrates how one steroid analyte can be contribute toward multiple homologous series in the HC[H] class.

Studying the ionization of steroid standards demonstrates that the intensity patterns seen in the DBE versus carbon number plots in Figure 4 likely arise due to steroid, or more widely, triterpenoid contributions. However, the six steroid standards investigated each demonstrated the formation of 3–4 major, along with various minor, ion species via APPI, and thus the DBE versus carbon number plots have more complex interpretations than what might have otherwise been assumed.

### 3.5 CID Fragmentation of Steroid Standard [M]^•+^ Ions

The isolation and CID fragmentation spectra for the steroid standards and corresponding peaks in the mid-Kawangware spectrum are given in Figures S.7–S.12 (SI). Fragment lists are also included in Tables S.11–S.16 for the six standard compounds in the SI. Interpretations for the key sterol and stanol CID fragments detected are provided in Figures S.13 and S.14 (SI). These either included the side chains on C_17_ and are hence strongly indicative of a specific compound, or they were representative of the generic steroid core. The former fragments occurred at different *m*/*z* for different steroids, whereas the steroid core fragments had diagnostic *m*/*z* values for sterols and stanols. The main fragments from the steroid standard CID spectra (Figures S.13 and S.14) have been indicated in Tables S.17 and S.18 (for the sterols and stanols, respectively), along with the presence or absence of these fragments in the corresponding mid-Kawangware CID spectra. Broadly, the main steroid fragments were detected in the mid-Kawangware CID spectra, and the three fragments missing from the suspected stigmasterol spectrum were only low intensity signals in the reference spectrum.

The comparisons provided in Tables S.17 and S.18 verify that the signals in the mid-Kawangware spectrum are indeed likely to represent steroids, either the standard reference compounds analyzed herein, or closely related isomers. This type of CID fragmentation analysis supports the hypothesis that the intense patterns in the HC, HC[H], O_1_, and O_1_[H] class DBE versus carbon number plots (Section 3.2.1) derived from the complex mixture data represent steroids, or more broadly, triterpenoids.

## 4 Conclusions

The untargeted analysis of organic sediment extracts from Nairobi River, using APPI–FT–ICR–MS, has provided insights into the anthropogenic contributions from the Kawangware and Kiambio slums, supporting previous targeted investigations. Compound classes with distinctive complex mixture patterns have provided pollution insights that were otherwise undetected for these environments, furthering the knowledge base of the impacts of urbanization on river sediments. In this way, FT–ICR–MS is particularly useful, because it can highlight potential compounds and chemical classes warranting further, targeted investigation.

Nairobi River was previously shown to be impacted by human sewage contributions thorough fecal steroid quantification work. Distinctive intense patterns in the sediment HC, HC[H], and O_1_ classes, in the APPI–FT–ICR–MS data herein, were consistent with steroid molecular formulas, including cholesterol and coprostanol. This interpretation was confirmed through evaluating the ionization behavior of a range of steroid standards via APPI, and through comparing the CID fragments of these compounds with those from the corresponding ions from an example sediment sample. The steroids investigated generally produced abundant radical molecular ions ([M]^•+^), and two abundant water-loss molecular ion species ([M–H_2_O]^•+^ and [M+H–H_2_O]^+^), among various other less intense species. Notably, pseudo molecular protonated species ([M+H]^+^) were not observed, which might be unexpected given that the intense “steroid” pattern in the complex mixture data was present for the O_1_[H] class. Other observations, including the formation of CH_4_-, H_2_-, and 4H-loss protonated species by steroids, can account for the O_1_[H] class features. While these investigations strongly support the steroid (or more broadly, triterpenoid) interpretation of the intense HC, HC[H], O_1_, and O_1_[H] class patterns, they also highlight the greater complexity of pattern interpretation in these data sets. Furthermore, the occurrence of this “steroid” pattern at higher oxygenation (O_2_/O_2_[H] and O_3_[H] classes) in the sediments could in part be accounted for by steroid ionization behavior, but likely also indicates higher oxygenated triterpenoids in these environments.

Notable patterns in the APPI–FT–ICR–MS S_1_ class data for the Nairobi sediments are indicative of pollution from thiophenic compounds, with more prominent benzothiophene series interpreted with a possible kerosene origin. These suspected thiophenic contributions are localized to the mid-slum sampling point for Kawangware, but are spread across the pre-, mid-, and post-slum sites for Kiambio, possibly reflecting Kiambio’s closer proximity to more urbanized areas and industrial developments. Other oxygenated sulfur-based classes had intense contributions from molecular formulas suspected to be surfactant-like compounds. The most intense of these signals, with greatest abundance associated with mid-slum sites, were observed in the O_2_S_1_[H] class, and these were hypothesized to be engine oil additives.

Other features of interest in the APPI–FT–ICR–MS sediment data were noted for the O_4_[H], N_1_O_2_[H], and N_1_O_3_[H] classes. The patterns in the N_1_O_2_[H] and N_1_O_3_[H] classes were interpreted in relation to sphingolipids with a bacterial origin, either due to the presence of persistent organic pollutants (Sphingomonas) or owing to sewage inputs (Bacteroidetes).

## Supporting Information Available

Steroid standard details, data recalibration tables, steroid standard peak lists (broadband and CID spectra), mass spectrum overviews, additional DBE plots, and fragmentation diagrams (PDF)
