# Supplementary material for: Sewage and Organic Pollution Compounds in Nairobi River Urban Sediments Characterized by Fourier Transform Ion Cyclotron Resonance Mass Spectrometry (FT–ICR–MS)
Source: J Am Soc Mass Spectrom. 2024 Sep 3;35(10):2376–89. doi: 10.1021/jasms.4c00229 (PMC11450967; doi:10.1021/jasms.4c00229)
Supplement: Supplementary file 1 — js4c00229_si_001.pdf [file js4c00229_si_001.pdf]

Supporting Information

Sewage and organic pollution compounds in Nairobi River urban sediments characterized by Fourier transform ion cyclotron resonance mass spectrometry (FT-ICR-MS)

Rory P. Downham[a], Christopher H. Vane[b], Benedict Gannon[a], Lydia A. Olaka[c], and Mark P. Barrow[a]*

a. Department of Chemistry, University of Warwick, Coventry, CV4 7AL, UK
b. British Geological Survey, Organic Geochemistry Facility, Keyworth, Nottingham, NG12 5GG, UK
c. Technical University of Kenya, Department of Geoscience and Environment, P.O. Box 52428-00200, Nairobi, Kenya

*Mark P. Barrow - Department of Chemistry, University of Warwick, Coventry, CV4 7AL, UK; orcid.org/0000-0002-6474-5357; Email: m.p.barrow@warwick.ac.uk

Supplementary Tables

Table S.1: Details for the steroid standards.

Compound name	Formula	CAS no.	Structure	Product details
Cholesterol (5-Cholesten-3β-ol)	$C_{27}H_{46}O$	57-88-5	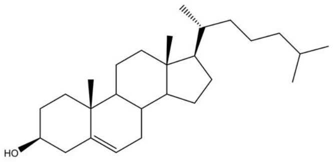	CRS, European Pharmacopoeia C2155000
β-Sitosterol (Stigmast-5-en-3β-ol)	$C_{29}H_{50}O$	83-46-5	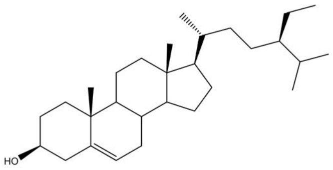	Avanti Polar Lipids 700095
Stigmasterol (Stigmasta-5,22-dien-3β-ol)	$C_{29}H_{48}O$	83-48-7	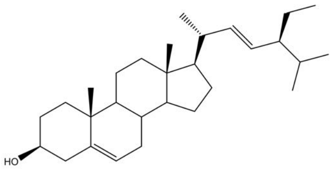	Avanti Polar Lipids 700062(P)
Coprostanol (5β-Cholestan-3β-ol)	$C_{27}H_{48}O$	360-68-9	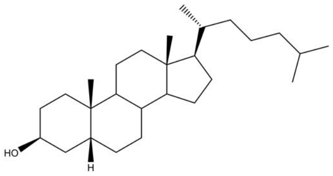	Sigma Aldrich C7578
Cholestanol (5α-Cholestan-3β-ol)	$C_{27}H_{48}O$	80-97-7	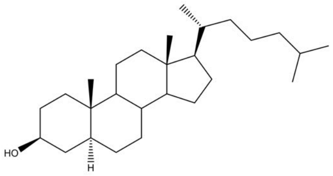	Avanti Polar Lipids 700064(P)
5α-Sitostanol (5α-Stigmastan-3β-ol	$C_{29}H_{52}O$	83-45-4	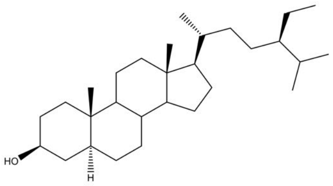	Avanti Polar Lipids 700121(P)

Table S.2: Homologous series for recalibrating sediment APPI-FTICR mass spectra.

Ion formulae (homologous series)	m/z range
$[C_{21}H_{32}+H]^+$ to $[C_{56}H_{102}+H]^+$	285.257677 to 775.805429
$[C_{19}H_{30}+H]^+$ to $[C_{57}H_{106}+H]^+$	259.242027 to 791.836729
$[C_{21}H_{42}O_1+H]^+$ to $[C_{53}H_{106}O_1+H]^+$	311.330842 to 759.831644

Table S.3: Steroid standard broadband recalibration lists

Compound	Ion formula	Ion mass (calculated)
Cholesterol	$[C_{27}H_{46}O]^{\bullet+}$	386.354318
	$[C_{27}H_{42}O+H]^+$	383.330842
	$[C_{27}H_{44}+H]^+$	369.351578
	$[C_{27}H_{44}]^{\bullet+}$	368.343752
β -Sitosterol	$[C_{29}H_{50}O]^{\bullet+}$	414.385617
	$[C_{29}H_{48}+H]^+$	397.382878
	$[C_{29}H_{46}O+H]^+$	411.362142
	$[C_{29}H_{48}]^{\bullet+}$	396.375053
Stigmasterol	$[C_{29}H_{48}O]^{\bullet+}$	412.369967
	$[C_{29}H_{46}+H]^+$	395.367228
	$[C_{29}H_{46}]^{\bullet+}$	394.359402
	$[C_{28}H_{42}+H]^+$	379.335927
Coprostanol and cholestanol	$[C_{27}H_{48}O]^{\bullet+}$	388.369967
	$[C_{27}H_{46}+H]^+$	371.367228
	$[C_{26}H_{44}O+H]^+$	373.346492
	$[C_{26}H_{42}+H]^+$	355.335927
5α-Sitostanol	$[C_{29}H_{52}O]^{\bullet+}$	416.401267
	$[C_{28}H_{48}O+H]^+$	401.377792
	$[C_{29}H_{50}+H]^+$	399.398528
	$[C_{28}H_{46}+H]^+$	383.367228

Table S.4: Steroid standard CID recalibration lists.

Compound	Ion formula (simplified)	Ion mass (calculated)
Cholesterol	$[C_{27}H_{46}O]^+$	386.354318
	$[C_{27}H_{44}]^+$	368.343753
	$[C_{26}H_{43}O]^+$	371.330842
	$[C_{26}H_{41}]^+$	353.320278
	$[C_{24}H_{38}]^+$	326.296803
	$[C_{22}H_{37}]^+$	301.288978
	$[C_{19}H_{27}]^+$	255.210727
	$[C_{16}H_{23}O]^+$	231.174342
	$[C_{16}H_{21}]^+$	213.163777
	$[C_{12}H_{18}O]^+$	178.135217
β -Sitosterol	$[C_{29}H_{50}O]^+$	414.385617
	$[C_{28}H_{47}O]^+$	399.362142
	$[C_{28}H_{45}]^+$	381.351577
	$[C_{26}H_{42}]^+$	354.328102
	$[C_{24}H_{41}]^+$	329.320277
	$[C_{19}H_{29}O]^+$	273.221292
	$[C_{19}H_{27}]^+$	255.210727
	$[C_{16}H_{23}O]^+$	231.174342
	$[C_{16}H_{21}]^+$	213.163777
Stigmasterol	$[C_{29}H_{48}O]^+$	412.369967
	$[C_{29}H_{46}]^+$	394.359402
	$[C_{28}H_{43}]^+$	379.335927
	$[C_{19}H_{27}O]^+$	271.205641
	$[C_{15}H_{17}]^+$	197.132477
	$[C_{14}H_{17}]^+$	185.132477
Coprostanol and cholestanol	$[C_{27}H_{48}O]^+$	388.369967
	$[C_{27}H_{46}]^+$	370.359402
	$[C_{26}H_{43}]^+$	355.335927
	$[C_{19}H_{34}]^+$	262.265502
	$[C_{16}H_{25}O]^+$	233.189991
	$[C_{16}H_{23}]^+$	215.179427
5α-Sitostanol	$[C_{29}H_{52}O]^+$	416.401267
	$[C_{28}H_{49}O]^+$	401.377792
	$[C_{28}H_{47}]^+$	383.367228
	$[C_{21}H_{38}]^+$	290.296802
	$[C_{16}H_{25}O]^+$	233.189991
	$[C_{16}H_{23}]^+$	215.179427

Table S.5. Cholesterol monoisotopic ions detected via broadband APPI-FTICR MS (in propanol and toluene, but not dichloromethane), with a relative intensity > 1%.

**Peak m/z**	**Relative intensity, %**	**Assigned m/z**	**Ion formula**	**Interpretation**	**Detected by van Agthoven et al.?[1]**
369.35158	100	369.351578	$[C_{27}H_{44}+H]^{+}$	$[M+H–H_2O]^{+}$	Y
368.34375	92.96	368.343753	$[C_{27}H_{44}]^{\bullet+}$	$[M–H_2O]^{\bullet+}$	Y
386.35431	60.77	386.354318	$[C_{27}H_{46}O]^{\bullet+}$	$[M]^{\bullet+}$	Y
385.34651	7.74	385.346492	$[C_{27}H_{44}O+H]^{+}$	$[M+H–H_2]^{+}$	Y
371.33086	7.49	371.330842	$[C_{26}H_{42}O+H]^{+}$	$[M+H–CH_4]^{+}$	Y
383.33085	6.89	383.330842	$[C_{27}H_{42}O+H]^{+}$	$[M+H–4H]^{+}$	Y
384.33868	4.14	384.338667	$[C_{27}H_{44}O]^{\bullet+}$	$[M–H_2]^{\bullet+}$	Y
353.32031	3.78	353.320278	$[C_{26}H_{40}+H]^{+}$		Y
367.33596	2.55	367.335928	$[C_{27}H_{42}+H]^{+}$	$[M+H–H_2O–H_2]^{+}$	Y
301.28903	2.40	301.288978	$[C_{22}H_{36}+H]^{+}$		Y
177.16386	1.92	177.163777	$[C_{13}H_{20}+H]^{+}$	Solvent background ion	N
401.34142	1.11	401.341407	$[C_{27}H_{44}O_2+H]^{+}$	$[M+H+O–H_2]^{+}$	Y
366.32813	1.09	366.328103	$[C_{27}H_{42}]^{\bullet+}$	$[M–H_2O–H_2]^{\bullet+}$	Y
382.32302	1.05	382.323017	$[C_{27}H_{42}O]^{\bullet+}$	$[M–4H]^{\bullet+}$	Y

Table S.6. β-sitosterol monoisotopic ions detected via broadband APPI-FTICR MS (in propanol and toluene), with a relative intensity > 1%.

Peak m/z	Relative intensity, %	Assigned m/z	Ion formula	Interpretation
397.38288	100.00	397.382878	$[C_{29}H_{48}+H]^+$	$[M+H–H_2O]^+$
396.37505	81.18	396.375053	$[C_{29}H_{48}]^{\bullet+}$	$[M–H_2O]^{\bullet+}$
414.38561	71.19	414.385618	$[C_{29}H_{50}O]^{\bullet+}$	$[M]^{\bullet+}$
411.36215	12.94	411.362143	$[C_{29}H_{46}O+H]^+$	$[M+H–4H]^+$
413.37778	12.43	413.377793	$[C_{29}H_{48}O+H]^+$	$[M+H–H_2]^+$
412.36996	7.22	412.369968	$[C_{29}H_{48}O]^{\bullet+}$	$[M–H_2]^{\bullet+}$
487.42978	6.12	487.429828	$[C_{36}H_{54}+H]^+$	Unknown
399.36215	5.54	399.362143	$[C_{28}H_{46}O+H]^+$	$[M+H–CH_4]^+$
443.38835	4.23	443.388357	$[C_{30}H_{50}O_2+H]^+$	Unknown
381.35157	4.06	381.351578	$[C_{28}H_{44}+H]^+$	$[M+H–CH_4–H_2O]^+$
503.42472	3.86	503.424743	$[C_{36}H_{54}O+H]^+$	Unknown
429.37270	3.17	429.372707	$[C_{29}H_{48}O_2+H]^+$	Contaminant
177.16376	2.83	177.163777	$[C_{13}H_{20}+H]^+$	Solvent background ion
395.36725	2.80	395.367228	$[C_{29}H_{46}+H]^+$	$[M+H–H_2–H_2O]^+$
410.35433	1.61	410.354318	$[C_{29}H_{46}O]^{\bullet+}$	$[M–4H]^{\bullet+}$
383.36722	1.58	383.367228	$[C_{28}H_{46}+H]^+$	$[M+H–CH_4O]^+$
329.32025	1.57	329.320278	$[C_{24}H_{40}+H]^+$	
427.35704	1.26	427.357057	$[C_{29}H_{46}O_2+H]^+$	Contaminant
428.36485	1.22	428.364882	$[C_{29}H_{48}O_2]^{\bullet+}$	$[M+O–H_2]^{\bullet+}$
394.35940	1.18	394.359403	$[C_{29}H_{46}]^{\bullet+}$	$[M–H_2O–H_2]^{\bullet+}$
485.43527	1.09	485.435307	$[C_{33}H_{56}O_2+H]^+$	Unknown
382.35940	1.04	382.359403	$[C_{28}H_{46}]^{\bullet+}$	$[M–CH_4O]^{\bullet+}$
489.40907	1.00	489.409093	$[C_{35}H_{52}O+H]^+$	Unknown

Table S.7: Stigmasterol monoisotopic ions detected via broadband APPI-FTICR MS (in propanol and toluene), with a relative intensity > 1%.

**Peak m/z**	**Relative intensity, %**	**Assigned m/z**	**Ion formula**	**Interpretation**
412.36996	100.00	412.369968	$[C_{29}H_{48}O]^{\bullet+}$	$[M]^{\bullet+}$
395.36723	59.01	395.367228	$[C_{29}H_{46}+H]^{+}$	$[M+H–H_2O]^{+}$
394.35942	42.64	394.359403	$[C_{29}H_{46}]^{\bullet+}$	$[M–H_2O]^{\bullet+}$
409.34652	8.62	409.346492	$[C_{29}H_{44}O+H]^{+}$	$[M+H–4H]^{+}$
369.31516	7.92	369.315192	$[C_{26}H_{40}O+H]^{+}$	$[M+H–C_3H_8]^{+}$
300.24459	7.67	300.244767	$[C_{21}H_{32}O]^{\bullet+}$	$[M–C_8H_{16}]^{\bullet+}$
411.36217	5.89	411.362143	$[C_{29}H_{46}O+H]^{+}$	$[M+H–H_2]^{+}$
379.33592	4.14	379.335928	$[C_{28}H_{42}+H]^{+}$	$[M+H–CH_4–H_2O]^{+}$
255.21051	4.12	255.210727	$[C_{19}H_{26}+H]^{+}$	$[M+H–C_{10}H_{22}O]^{+}$
351.30456	4.08	351.304628	$[C_{26}H_{38}+H]^{+}$	$[M+H–C_3H_8–H_2O]^{+}$
271.20544	4.02	271.205642	$[C_{19}H_{26}O+H]^{+}$	$[M+H–C_{10}H_{22}]^{+}$
397.34651	3.77	397.346492	$[C_{28}H_{44}O+H]^{+}$	$[M+H–CH_4]^{+}$
314.26026	2.95	314.260417	$[C_{22}H_{34}O]^{\bullet+}$	$[M–C_7H_{14}]^{\bullet+}$
410.35436	2.94	410.354318	$[C_{29}H_{46}O]^{\bullet+}$	$[M–H_2]^{\bullet+}$
311.27317	2.92	311.273328	$[C_{23}H_{34}+H]^{+}$	
427.35713	2.87	427.357057	$[C_{29}H_{46}O_2+H]^{+}$	Contaminant
410.39074	2.81	410.390703	$[C_{30}H_{50}]^{\bullet+}$	Contaminant
299.23676	2.51	299.236942	$[C_{21}H_{30}O+H]^{+}$	
282.23401	1.67	282.234202	$[C_{21}H_{30}]^{\bullet+}$	
407.33089	1.66	407.330842	$[C_{29}H_{42}O+H]^{+}$	
393.35161	1.54	393.351578	$[C_{29}H_{44}+H]^{+}$	
270.19761	1.46	270.197817	$[C_{19}H_{26}O]^{\bullet+}$	
272.21327	1.42	272.213467	$[C_{19}H_{28}O]^{\bullet+}$	
297.2575	1.37	297.257677	$[C_{22}H_{32}+H]^{+}$	
273.22109	1.15	273.221292	$[C_{19}H_{28}O+H]^{+}$	
398.35431	1.12	398.354318	$[C_{28}H_{46}O]^{\bullet+}$	
381.3516	1.11	381.351578	$[C_{28}H_{44}+H]^{+}$	
408.33872	1.09	408.338667	$[C_{29}H_{44}O]^{\bullet+}$	
396.3387	1.06	396.338667	$[C_{28}H_{44}O]^{\bullet+}$	

Table S.8. Coprostanol monoisotopic ions detected via broadband APPI-FTICR MS (in propanol and toluene), with a relative intensity > 1%.

Peak m/z	Relative intensity, %	Assigned m/z	Ion formula	Interpretation
388.36997	100.00	388.36997	$[C_{27}H_{48}O]^{\bullet+}$	$[M]^{\bullet+}$
373.34649	46.33	373.34649	$[C_{26}H_{44}O+H]^+$	$[M+H–CH_4]^+$
371.36723	45.58	371.36723	$[C_{27}H_{46}+H]^+$	$[M+H–H_2O]^+$
355.33593	32.43	355.33593	$[C_{26}H_{42}+H]^+$	$[M+H–CH_4–H_2O]^+$
177.16385	30.21	177.16378	$[C_{13}H_{20}+H]^+$	Solvent background ion
370.35941	26.19	370.3594	$[C_{27}H_{46}]^{\bullet+}$	$[M–H_2O]^{\bullet+}$
387.36213	10.65	387.36214	$[C_{27}H_{46}O+H]^+$	$[M+H–H_2]^+$
369.35159	6.76	369.35158	$[C_{27}H_{44}+H]^+$	$[M+H–H_2O–H_2]^+$
215.17950	6.23	215.17943	$[C_{16}H_{22}+H]^+$	
435.38326	5.80	435.38327	$[C_{28}H_{50}O_3+H]^+$	Unknown
461.41400	4.17	461.41418	$[C_{34}H_{52}+H]^+$	Unknown
383.36726	3.66	383.36723	$[C_{28}H_{46}+H]^+$	Unknown
403.35702	3.58	403.35706	$[C_{27}H_{46}O_2+H]^+$	$[M+H+O–H_2]^+$
464.43764	3.43	464.43765	$[C_{34}H_{56}]^{\bullet+}$	Unknown
385.34649	3.26	385.34649	$[C_{27}H_{44}O+H]^+$	$[M+H–4H]^+$
421.36758	2.54	421.36762	$[C_{27}H_{48}O_3+H]^+$	$[M+H+O_2]^+$
493.40377	2.30	493.40401	$[C_{34}H_{52}O_2+H]^+$	Unknown
386.35432	2.17	386.35432	$[C_{27}H_{46}O]^{\bullet+}$	$[M–H_2]^{\bullet+}$
233.19007	2.03	233.18999	$[C_{16}H_{24}O+H]^+$	
331.29951	2.00	331.29954	$[C_{23}H_{38}O+H]^+$	
400.37001	1.99	400.36997	$[C_{28}H_{48}O]^{\bullet+}$	Unknown
417.37267	1.96	417.37271	$[C_{28}H_{48}O_2+H]^+$	Unknown
288.28119	1.77	288.28115	$[C_{21}H_{36}]^{\bullet+}$	
199.11180	1.75	199.11174	$[C_{14}H_{14}O+H]^+$	Solvent background ion
462.42185	1.69	462.422	$[C_{34}H_{54}]^{\bullet+}$	Unknown
479.38806	1.62	479.38836	$[C_{33}H_{50}O_2+H]^+$	Unknown
402.38572	1.53	402.38562	$[C_{28}H_{50}O]^{\bullet+}$	Unknown
447.39862	1.51	447.39853	$[C_{33}H_{50}+H]^+$	Unknown
429.40930	1.50	429.40909	$[C_{30}H_{52}O+H]^+$	Unknown
201.16386	1.39	201.16378	$[C_{15}H_{20}+H]^+$	
185.13256	1.25	185.13248	$[C_{14}H_{16}+H]^+$	Solvent background ion
198.10399	1.21	198.10392	$[C_{14}H_{14}O]^{\bullet+}$	Solvent background ion
445.40414	1.15	445.40401	$[C_{30}H_{52}O_2+H]^+$	Unknown
367.33592	1.13	367.33593	$[C_{27}H_{42}+H]^+$	
219.21082	1.09	219.21073	$[C_{16}H_{26}+H]^+$	Contaminant
183.11693	1.00	183.11683	$[C_{14}H_{14}+H]^+$	Solvent background ion

Table S.9. Cholestanol monoisotopic ions detected via broadband APPI-FTICR MS (in propanol and toluene), with a relative intensity > 1%.

Peak m/z	Relative intensity, %	Assigned m/z	Ion formula	Interpretation
388.36997	100.00	388.369968	$[C_{27}H_{48}O]^{\bullet+}$	$[M]^{\bullet+}$
371.36723	19.49	371.367228	$[C_{27}H_{46}+H]^{+}$	$[M+H–H_2O]^{+}$
373.34649	11.12	373.346492	$[C_{26}H_{44}O+H]^{+}$	$[M+H–CH_4]^{+}$
387.36214	6.24	387.362143	$[C_{27}H_{46}O+H]^{+}$	$[M+H–H_2]^{+}$
355.33593	4.02	355.335928	$[C_{26}H_{42}+H]^{+}$	$[M+H–CH_4–H_2O]^{+}$
370.35940	2.28	370.359403	$[C_{27}H_{46}]^{\bullet+}$	$[M–H_2O]^{\bullet+}$
177.16379	1.32	177.163777	$[C_{13}H_{20}+H]^{+}$	Solvent background ion

Table S.10. 5α-Sitostanol monoisotopic ions detected via broadband APPI-FTICR MS (in propanol and toluene), with a relative intensity > 1%.

Peak m/z	Relative intensity, %	Assigned m/z	Ion formula	Interpretation
416.40126	100.00	416.401268	$[C_{29}H_{52}O]^{\bullet+}$	$[M]^{\bullet+}$
399.39853	14.74	399.398528	$[C_{29}H_{50}+H]^{+}$	$[M+H–H_2O]^{+}$
530.46924	12.08	530.469347	$[C_{35}H_{62}O_3]^{\bullet+}$	Contaminant
401.37780	10.10	401.377793	$[C_{28}H_{48}O+H]^{+}$	$[M+H–CH_4]^{+}$
415.39344	7.77	415.393443	$[C_{29}H_{50}O+H]^{+}$	$[M+H–H_2]^{+}$
177.16379	4.42	177.163777	$[C_{13}H_{20}+H]^{+}$	Solvent background ion
383.36723	4.04	383.367228	$[C_{28}H_{46}+H]^{+}$	$[M+H–CH_4–H_2O]^{+}$
447.34686	3.05	447.346886	$[C_{28}H_{46}O_4+H]^{+}$	Contaminant
398.39072	2.78	398.390703	$[C_{29}H_{50}]^{\bullet+}$	$[M–H_2O]^{\bullet+}$
402.38562	2.48	402.385618	$[C_{28}H_{50}O]^{\bullet+}$	
413.37778	1.55	413.377793	$[C_{29}H_{48}O+H]^{+}$	
474.40667	1.27	474.406747	$[C_{31}H_{54}O_3]^{\bullet+}$	Contaminant
397.38287	1.08	397.382878	$[C_{29}H_{48}+H]^{+}$	
414.38563	1.00	414.385618	$[C_{29}H_{50}O]^{\bullet+}$	

Table S.11: Ion formula assignments for the standard cholesterol CID spectrum. Interpretations marked are based on work by West and Reid[2].*

Cholesterol standard fragmentation mass spectrum					Interpretation	Detected in Kawangware CID spectrum, for isolation at m/z 386.35? (rel. inten., %)
**Peak m/z**	**Rel. inten., %**	**Assigned m/z**	**Ion formula (simplified)**	**Electron species**		
386.35435	100.00	386.354318	$[C_{27}H_{46}O]^+$	Odd	$[M]^{+\bullet}$	Y (37.41)
231.17433	72.49	231.174342	$[C_{16}H_{23}O]^+$	Even	$C_{11}H_{23}$ loss*	Y (79.02)
301.28897	64.66	301.288978	$[C_{22}H_{37}]^+$	Even	C_5H_9O loss*	Y (29.73)
371.33086	56.45	371.330842	$[C_{26}H_{43}O]^+$	Even	CH_3 loss*	Y (85.56)
368.34376	35.53	368.343753	$[C_{27}H_{44}]^+$	Odd	H_2O loss*	Y (12.03)
213.16377	23.39	213.163777	$[C_{16}H_{21}]^+$	Even	H_2O and $C_{11}H_{23}$ losses	Y (59.02)
353.32027	21.43	353.320278	$[C_{26}H_{41}]^+$	Even	H_2O and CH_3 losses	Y (35.38)
255.21072	21.23	255.210727	$[C_{19}H_{27}]^+$	Even	H_2O and C_8H_{17} loss*	Y (14.85)
273.22129	20.29	273.221292	$[C_{19}H_{29}O]^+$	Even	C_8H_{17} loss*	Y (14.94)
229.19507	13.65	229.195077	$[C_{17}H_{25}]^+$	Even	$C_{10}H_{21}O$ loss*	Y (14.65)
326.29680	13.34	326.296803	$[C_{24}H_{38}]^+$	Odd		Y (5.53)
246.19781	13.02	246.197817	$[C_{17}H_{26}O]^+$	Odd		Y (20.81)
228.18724	13.01	228.187252	$[C_{17}H_{24}]^+$	Odd		Y (9.66)
343.29953	8.99	343.299542	$[C_{24}H_{39}O]^+$	Even		N
178.13523	7.40	178.135217	$[C_{12}H_{18}O]^+$	Odd		N
161.13249	7.29	161.132477	$[C_{12}H_{17}]^+$	Even		Y (19.99)
327.30463	7.25	327.304628	$[C_{24}H_{39}]^+$	Even		Y (13.02)
245.18998	6.93	245.189992	$[C_{17}H_{25}O]^+$	Even		Y (7.55)
227.17942	6.46	227.179427	$[C_{17}H_{23}]^+$	Even		Y (7.25)
300.28112	5.74	300.281153	$[C_{22}H_{36}]^+$	Odd		N
232.18215	5.56	232.182167	$[C_{16}H_{24}O]^+$	Odd		Y (17.79)
313.28896	5.30	313.288978	$[C_{23}H_{37}]^+$	Even		N
173.13249	5.01	173.132477	$[C_{13}H_{17}]^+$	Even		Y (8.37)
301.25257	4.80	301.252592	$[C_{21}H_{33}O]^+$	Even		N
160.12467	4.57	160.124652	$[C_{12}H_{16}]^+$	Odd		N
357.31526	4.51	357.315192	$[C_{25}H_{41}O]^+$	Even		N
189.16378	4.28	189.163777	$[C_{14}H_{21}]^+$	Even	$C_{13}H_{25}O$ loss*	Y (8.65)
175.14814	3.96	175.148127	$[C_{13}H_{19}]^+$	Even		Y (8.57)
314.29681	3.96	314.296803	$[C_{23}H_{38}]^+$	Odd		Y (4.73)
159.11683	3.62	159.116827	$[C_{12}H_{15}]^+$	Even		Y (4.56)
163.14813	3.53	163.148127	$[C_{12}H_{19}]^+$	Even		Y (5.66)
199.14812	3.40	199.148127	$[C_{15}H_{19}]^+$	Even	$C_{12}H_{25}$ and H_2O losses	Y (13.80)
325.28901	3.31	325.288978	$[C_{24}H_{37}]^+$	Even		Y (3.69)
187.14811	3.28	187.148127	$[C_{14}H_{19}]^+$	Even		Y (7.20)
339.30460	3.18	339.304628	$[C_{25}H_{39}]^+$	Even		N

(Table S.11 continued…)

206.20288	3.09	206.202902	$[C_{15}H_{26}]^+$	Odd		N
203.14301	3.04	203.143042	$[C_{14}H_{19}O]^+$	Even		Y (4.04)
145.10121	3.03	145.101177	$[C_{11}H_{13}]^+$	Even		N
214.17160	2.74	214.171602	$[C_{16}H_{22}]^+$	Odd		Y (12.50)
147.11684	2.72	147.116827	$[C_{11}H_{15}]^+$	Even		N
201.16377	2.54	201.163777	$[C_{15}H_{21}]^+$	Even		Y (11.87)
299.27325	2.45	299.273328	$[C_{22}H_{35}]^+$	Even		N
260.21347	2.41	260.213467	$[C_{18}H_{28}O]^+$	Odd		Y (5.42)
174.14030	2.36	174.140302	$[C_{13}H_{18}]^+$	Odd		N
185.13248	2.18	185.132477	$[C_{14}H_{17}]^+$	Even		Y (9.81)
215.17938	2.18	215.179427	$[C_{16}H_{23}]^+$	Even		Y (12.67)
316.31241	2.18	316.312453	$[C_{23}H_{40}]^+$	Odd		Y (31.75)
149.13251	2.11	149.132477	$[C_{11}H_{17}]^+$	Even		Y (2.90)
283.24201	2.07	283.242027	$[C_{21}H_{31}]^+$	Even		N
171.11688	2.05	171.116827	$[C_{13}H_{15}]^+$	Even		Y (4.19)
285.25783	1.98	285.257677	$[C_{21}H_{33}]^+$	Even		N
191.17945	1.95	191.179427	$[C_{14}H_{23}]^+$	Even		Y (5.35)
135.11683	1.89	135.116827	$[C_{10}H_{15}]^+$	Even		N
242.20286	1.77	242.202902	$[C_{18}H_{26}]^+$	Odd		N
340.31251	1.74	340.312453	$[C_{25}H_{40}]^+$	Odd		N
158.10902	1.73	158.109002	$[C_{12}H_{14}]^+$	Odd		N
247.20563	1.72	247.205642	$[C_{17}H_{27}O]^+$	Even		N
200.15589	1.55	200.155952	$[C_{15}H_{20}]^+$	Odd		N
164.11958	1.52	164.119567	$[C_{11}H_{16}O]^+$	Odd		N
172.12469	1.50	172.124652	$[C_{13}H_{16}]^+$	Odd		N

Table S.12: Ion formula assignments for the β-sitosterol CID spectrum.

**β-sitosterol standard fragmentation mass spectrum**					**Interpretation**	**Detected in Kawangware CID spectrum, for isolation at m/z 414.39? (rel. inten., %)**
**Peak m/z**	**Rel. inten., %**	**Assigned m/z**	**Ion formula (simplified)**	**Electron species**		
213.16379	100.00	213.163777	$[C_{16}H_{21}]^+$	Even	H_2O and $C_{13}H_{27}$ losses	Y (45.65)
231.17434	95.30	231.174342	$[C_{16}H_{23}O]^+$	Even	$C_{13}H_{27}$ loss	Y (43.32)
399.36217	59.23	399.362143	$[C_{28}H_{47}O]^+$	Even	CH_3 loss	Y (100)
329.32025	48.75	329.320278	$[C_{24}H_{41}]^+$	Even	C_5H_9O loss	Y (7.50)
381.35160	29.20	381.351578	$[C_{28}H_{45}]^+$	Even	H_2O and CH_3 loss	Y (29.03)
255.21071	25.73	255.210727	$[C_{19}H_{27}]^+$	Even	H_2O and $C_{10}H_{21}$ losses	Y (12.89)
273.22127	24.81	273.221292	$[C_{19}H_{29}O]^+$	Even	$C_{10}H_{21}$ loss	Y (9.71)
414.38566	22.85	414.385618	$[C_{29}H_{50}O]^+$	Odd	$[M]^{\bullet+}$	Y (14.67)
228.18727	20.74	228.187252	$[C_{17}H_{24}]^+$	Odd		Y (8.69)
396.37508	20.31	396.375053	$[C_{29}H_{48}]^+$	Odd	H_2O loss	Y (4.47)
246.19779	19.80	246.197817	$[C_{17}H_{26}O]^+$	Odd		Y (19.30)
245.18997	15.44	245.189992	$[C_{17}H_{25}O]^+$	Even		Y (3.09)
229.19508	15.16	229.195077	$[C_{17}H_{25}]^+$	Even		Y (10.75)
227.17944	14.78	227.179427	$[C_{17}H_{23}]^+$	Even		Y (7.89)
354.32808	13.14	354.328103	$[C_{26}H_{42}]^+$	Odd		N
355.33593	10.65	355.335928	$[C_{26}H_{43}]^+$	Even		Y (15.41)
203.14305	10.28	203.143042	$[C_{14}H_{19}O]^+$	Even		Y (3.94)
185.13250	8.94	185.132477	$[C_{14}H_{17}]^+$	Even		Y (4.14)
178.13524	8.92	178.135217	$[C_{12}H_{18}O]^+$	Odd		N
214.17161	8.79	214.171602	$[C_{16}H_{22}]^+$	Odd		Y (5.58)
187.14815	8.35	187.148127	$[C_{14}H_{19}]^+$	Even		Y (7.22)
173.13250	8.23	173.132477	$[C_{13}H_{17}]^+$	Even		Y (7.98)
215.17943	8.08	215.179427	$[C_{16}H_{23}]^+$	Even	$C_{13}H_{25}$ and H_2O losses	Y (5.32)
161.13250	8.07	161.132477	$[C_{12}H_{17}]^+$	Even		Y (10.50)
199.14816	7.59	199.148127	$[C1_5H_{19}]^+$	Even	$C_{14}H_{29}$ and H_2O losses	Y (11.23)
201.16381	7.37	201.163777	$[C_{15}H_{21}]^+$	Even	$C_{14}H_{27}$ and H_2O losses	Y (7.36)
175.14816	7.06	175.148127	$[C_{13}H_{19}]^+$	Even		Y (4.91)
189.16381	6.91	189.163777	$[C_{14}H_{21}]^+$	Even		Y (6.00)
203.17943	6.56	203.179427	$[C_{15}H_{23}]^+$	Even		Y (7.48)
371.33080	6.56	371.330842	$[C_{26}H_{43}O]^+$	Even		N
232.18217	5.95	232.182167	$[C_{16}H_{24}O]^+$	Odd		Y (5.41)
328.31237	5.53	328.312453	$[C_{24}H_{40}]^+$	Odd		N
341.32029	5.15	341.320278	$[C_{25}H_{41}]^+$	Even		N
159.11685	4.65	159.116827	$[C_{12}H_{15}]^+$	Even		Y (4.93)
342.32803	4.59	342.328103	$[C_{25}H_{42}]^+$	Odd		Y (3.51)

(Table S.12 continued…)

353.32032	4.56	353.320278	$[C_{26}H_{41}]^+$	Even		N
217.19508	4.36	217.195077	$[C_{16}H_{25}]^+$	Even		Y (3.87)
241.19506	3.81	241.195077	$[C_{18}H_{25}]^+$	Even		Y (4.18)
171.11685	3.79	171.116827	$[C_{13}H_{15}]^+$	Even		Y (6.24)
186.14033	3.75	186.140302	$[C_{14}H_{18}]^+$	Odd		N
231.21073	3.42	231.210727	$[C_{17}H_{27}]^+$	Even		Y (2.69)
260.21343	3.17	260.213467	$[C_{18}H_{28}O]^+$	Odd		N
339.30467	2.83	339.304628	$[C_{25}H_{39}]^+$	Even		Y (2.77)
217.15870	2.71	217.158692	$[C_{15}H_{21}O]^+$	Even	$C_{14}H_{29}$ loss	Y (5.65)

Table S.13: Ion formula assignments for the stigmasterol CID spectrum.

**Stigmasterol standard fragmentation mass spectrum**					**Interpretation**	**Detected in Kawangware CID spectrum, for isolation at m/z 412.37? (rel. inten., %)**
**Peak m/z**	**Rel. inten., %**	**Assigned m/z**	**Ion formula (simplified)**	**Electron species**		
211.14812	100.00	211.148127	$[C_{16}H_{19}]^+$	Even	$C_{13}H_{29}O$ loss	Y (37.10)
379.33593	78.42	379.335928	$[C_{28}H_{43}]^+$	Even	H_2O and CH_3 losses	Y (45.73)
225.16377	45.36	225.163777	$[C_{17}H_{21}]^+$	Even	$C_{12}H_{27}O$ loss	Y (13.23)
226.17159	40.11	226.171602	$[C_{17}H_{22}]^+$	Odd	$C_{12}H_{26}O$ loss	Y (13.27)
197.13248	32.04	197.132477	$[C_{15}H_{17}]^+$	Even	$C_{14}H_{31}O$ loss	Y (13.92)
239.17941	23.55	239.179427	$[C_{18}H_{23}]^+$	Even		Y (8.88)
185.13248	22.37	185.132477	$[C_{14}H_{17}]^+$	Even		Y (12.73)
253.19506	22.01	253.195077	$[C_{19}H_{25}]^+$	Even	$C_{10}H_{21}$ and H_2O losses	Y (13.65)
169.10118	21.49	169.101177	$[C_{13}H_{13}]^+$	Even		Y (5.51)
199.14812	16.13	199.148127	$[C_{15}H_{19}]^+$	Even	$C_{14}H_{27}$ and H_2O losses	Y (10.54)
227.17941	13.88	227.179427	$[C_{17}H_{23}]^+$	Even		Y (8.54)
271.20562	13.53	271.205642	$[C_{19}H_{27}O]^+$	Even	$C_{10}H_{21}$ loss	Y (37.65)
213.16377	13.26	213.163777	$[C_{16}H_{21}]^+$	Even	H_2O and $C_{13}H_{25}$ losses	Y (15.20)
183.11683	13.11	183.116827	$[C_{14}H_{15}]^+$	Even		Y (5.91)
229.15868	12.50	229.158692	$[C_{16}H_{21}O]^+$	Even		Y (10.27)
171.11683	11.41	171.116827	$[C_{13}H_{15}]^+$	Even		Y (9.32)
244.18215	10.21	244.182167	$[C_{17}H_{24}O]^+$	Odd		N
394.35942	9.44	394.359402	$[C_{29}H_{46}]^+$	Odd	H_2O loss	Y (13.63)
397.34651	8.68	397.346492	$[C_{28}H_{45}O]^+$	Even	CH_3 loss	Y (32.25)
198.14030	8.60	198.140302	$[C_{15}H_{18}]^+$	Odd		N
186.14030	8.47	186.140302	$[C_{14}H_{18}]^+$	Odd		Y (5.80)
245.18997	6.36	245.189992	$[C_{17}H_{25}O]^+$	Even		Y (6.37)
212.15594	6.01	212.155952	$[C_{16}H_{20}]^+$	Odd		N
295.24200	5.79	295.242027	$[C_{22}H_{31}]^+$	Even		N
184.12465	5.69	184.124652	$[C_{14}H_{16}]^+$	Odd		N
281.22636	5.07	281.226377	$[C_{21}H_{29}]^+$	Even		N
412.36998	4.94	412.369968	$[C_{29}H_{48}O]^+$	Odd	$[M]^{\bullet+}$	Y (89.44)
243.17432	4.80	243.174342	$[C_{17}H_{23}O]^+$	Even		N
272.21342	3.76	272.213467	$[C_{19}H_{28}O]^+$	Odd		N
173.13249	3.61	173.132477	$[C_{13}H_{17}]^+$	Even		Y (6.82)
157.10119	3.37	157.101177	$[C_{12}H_{13}]^+$	Even		N
155.08554	3.25	155.085527	$[C_{12}H_{11}]^+$	Even		N
161.13249	3.09	161.132477	$[C_{12}H_{17}]^+$	Even		Y (8.54)
187.14813	3.04	187.148127	$[C_{14}H_{19}]^+$	Even		Y (6.74)
255.21071	3.04	255.210727	$[C_{19}H_{27}]^+$	Even	H_2O and $C_{10}H_{19}$ losses	Y (5.63)

(Table S.13 continued…)

257.18999	3.01	257.189992	$[C_{18}H_{25}O]^+$	Even		Y (6.62)
159.11683	2.92	159.116827	$[C_{12}H_{15}]^+$	Even		Y (5.73)
231.17433	2.76	231.174342	$[C_{16}H_{23}O]^+$	Even	$C_{13}H_{25}$ loss	Y (8.42)
258.19780	2.62	258.197817	$[C_{18}H_{26}O]^+$	Odd		N
214.17160	2.44	214.171602	$[C_{16}H_{22}]^+$	Odd		N
172.12465	2.30	172.124652	$[C_{13}H_{16}]^+$	Odd		N
217.15870	2.26	217.158692	$[C_{15}H_{21}O]^+$	Even	$C_{14}H_{27}$ loss	N
215.14304	2.20	215.143042	$[C_{15}H_{19}O]^+$	Even		N
201.16377	2.16	201.163777	$[C_{15}H_{21}]^+$	Even	$C_{14}H_{25}$ and H_2O losses	Y (11.08)
170.10901	2.14	170.109002	$[C_{13}H_{14}]^+$	Odd		N
241.19505	2.02	241.195077	$[C_{18}H_{25}]^+$	Even		N
326.29664	1.97	326.296802	$[C_{24}H_{38}]^+$	Odd		N
240.18723	1.92	240.187252	$[C_{18}H_{24}]^+$	Odd		N
174.14031	1.81	174.140302	$[C_{13}H_{18}]^+$	Odd		N
327.30460	1.75	327.304627	$[C_{24}H_{39}]^+$	Even	C_5H_9O loss	N
158.10903	1.66	158.109002	$[C_{12}H_{14}]^+$	Odd		N
349.28902	1.65	349.288978	$[C_{26}H_{37}]^+$	Even		N
201.12741	1.59	201.127392	$[C_{14}H_{17}O]^+$	Even		N
229.19508	1.47	229.195077	$[C_{17}H_{25}]^+$	Even		N
187.11174	1.46	187.111742	$[C_{13}H_{15}O]^+$	Even		N
353.32029	1.43	353.320278	$[C_{26}H_{41}]^+$	Even		N
200.15595	1.42	200.155952	$[C_{15}H_{20}]^+$	Odd		N

Table S.14: Ion formula assignments for the coprostanol CID spectrum.

**Coprostanol standard fragmentation mass spectrum**					**Interpretation**	**Detected in Kawangware CID spectrum, for isolation at m/z 388.37? (rel. inten., %)**
**Peak m/z**	**Rel. inten., %**	**Assigned m/z**	**Ion formula (simplified)**	**Electron species**		
373.34647	54.13	373.346492	$[C_{26}H_{45}O]^+$	Even	CH_3 loss	Y (100.00)
233.18998	29.99	233.189992	$[C_{16}H_{25}O]^+$	Even	$C_{11}H_{23}$ loss	Y (53.25)
388.36999	15.01	388.369968	$[C_{27}H_{48}O]^+$	Odd	$[M]^{\bullet+}$	Y (21.81)
355.33591	8.89	355.335928	$[C_{26}H_{43}]^+$	Even	H_2O and CH_3 losses	Y (23.80)
215.17943	8.68	215.179427	$[C_{16}H_{23}]^+$	Even	H_2O and $C_{11}H_{23}$ losses	Y (30.44)
262.26545	6.65	262.265503	$[C_{19}H_{34}]^+$	Odd	$C_8H_{14}O$ loss	Y (9.51)
288.28112	3.08	288.281153	$[C_{21}H_{36}]^+$	Odd	$C_6H_{12}O$ loss	Y (5.87)
370.35951	2.84	370.359403	$[C_{27}H_{46}]^+$	Odd	$[M–H_2O]^{\bullet+}$	Y (8.69)
201.16376	1.69	201.163777	$[C_{15}H_{21}]^+$	Even	$C_{12}H_{27}O$ loss	Y (2.77)

*Table S.15: Ion formula assignments for the cholestanol CID spectrum. The m/z values marked * were also detected in the coprostanol CID spectrum.*

Cholestanol standard fragmentation mass spectrum					Interpretation	Detected in Kawangware CID spectrum, for isolation at m/z 388.37? (rel. inten., %)
**Peak m/z**	**Rel. inten., %**	**Assigned m/z**	**Ion formula (simplified)**	**Electron species**		
373.34649	100.00	373.346492*	$[C_{26}H_{45}O]^+$	Even	CH_3 loss	Y (100.00)
233.19000	84.37	233.189992*	$[C_{16}H_{25}O]^+$	Even	$C_{11}H_{23}$ loss	Y (53.25)
215.17944	59.50	215.179427*	$[C_{16}H_{23}]^+$	Even	H_2O and $C_{11}H_{23}$ losses	Y (30.44)
262.26548	32.67	262.265503*	$[C_{19}H_{34}]^+$	Odd	$C_8H_{14}O$ loss	Y (9.51)
388.36999	27.24	388.369968*	$[C_{27}H_{48}O]^+$	Odd	$[M]^{\bullet+}$	Y (21.81)
355.33592	15.79	355.335928*	$[C_{26}H_{43}]^+$	Even	H_2O and CH_3 losses	Y (23.80)
216.18726	4.65	216.187252	$[C_{16}H_{24}]^+$	Odd	$C_{11}H_{22}$ and H_2O losses	Y (4.69)
234.19782	4.54	234.197817	$[C_{16}H_{26}O]^+$	Odd	$C_{11}H_{22}$ loss	Y (2.34)
288.28114	4.36	288.281153*	$[C_{21}H_{36}]^+$	Odd	$C_6H_{12}O$ loss	Y (5.87)
219.17435	3.74	219.174342	$[C_{15}H_{23}O]^+$	Even	$C_{12}H_{25}$ loss	Y (1.40)
370.35941	3.61	370.359403*	$[C_{27}H_{46}]^+$	Odd	$[M–H_2O]^{\bullet+}$	Y (8.69)
201.16378	2.52	201.163777*	$[C_{15}H_{21}]^+$	Even	$C_{12}H_{25}$ and H_2O losses	Y (2.77)
232.18217	2.52	232.182167	$[C_{16}H_{24}O]^+$	Odd		Y (0.87)
202.17161	1.85	202.171602	$[C_{15}H_{22}]^+$	Odd		Y (0.61)
178.13524	1.73	178.135217	$[C_{12}H_{18}O]^+$	Odd		Y (1.14)
331.29953	1.19	331.299542	$[C_{23}H_{38}O]^+$	Even		Y (1.60)
247.24203	0.81	247.242027	$[C_{18}H_{31}]^+$	Even		N
276.28111	0.79	276.281153	$[C_{20}H_{36}]^+$	Odd		Y (0.74)
260.24985	0.73	260.249852	$[C_{19}H_{32}]^+$	Odd		Y (0.51)
359.33081	0.72	359.330842	$[C_{25}H_{43}O]^+$	Even		N
229.19507	0.61	229.195077	$[C_{17}H_{25}]^+$	Even		Y (0.64)
341.32018	0.49	341.320278	$[C_{25}H_{41}]^+$	Even		N
230.20287	0.47	230.202902	$[C_{17}H_{26}]^+$	Odd		Y (0.70)
187.14814	0.37	187.148127	$[C_{14}H_{19}]^+$	Even		N
205.15873	0.34	205.158692	$[C_{14}H_{21}O]^+$	Even		N

Table S.16: Ion formula assignments for the 5α-sitostanol CID spectrum.

**5α-sitostanol standard fragmentation mass spectrum**					**Interpretation**	**Detected in Kawangware CID spectrum, for isolation at m/z 416.40? (rel. inten., %)**
**Peak m/z**	**Rel. inten., %**	**Assigned m/z**	**Ion formula (simplified)**	**Electron species**		
401.37780	100.00	401.377793	$[C_{28}H_{49}O]^+$	Even	CH_3 loss	Y (78.27)
233.18999	98.83	233.189992	$[C_{16}H_{25}O]^+$	Even	$C_{13}H_{27}$ loss	Y (60.89)
215.17943	56.86	215.179427	$[C_{16}H_{23}]^+$	Even	H_2O and $C_{13}H_{27}$ losses	Y (100.00)
290.29677	44.84	290.296803	$[C_{21}H_{38}]^+$	Odd	$C_8H_{14}O$ loss	Y (11.11)
416.40129	43.54	416.401268	$[C_{29}H_{52}O]^+$	Odd	$[M]^{\bullet+}$	Y (6.16)
383.36723	11.51	383.367228	$[C_{28}H_{47}]^+$	Even	H_2O and CH_3 losses	Y (76.84)
234.19782	10.42	234.197817	$[C_{16}H_{26}O]^+$	Odd	$C_{13}H_{26}$ loss	Y (2.97)
216.18725	8.31	216.187252	$[C_{16}H_{24}]^+$	Odd	H_2O and $C_{13}H_{26}$ losses	Y (6.83)
398.39071	5.04	398.390703	$[C_{29}H_{50}]^+$	Odd	H_2O loss	Y (4.33)
316.31243	4.90	316.312453	$[C_{23}H_{40}]^+$	Odd	$C_6H_{12}O$ loss	Y (5.59)
219.17436	4.90	219.174342	$[C_{15}H_{23}O]^+$	Even	$C_{14}H_{29}$ loss	Y (2.07)
232.18217	4.10	232.182167	$[C_{16}H_{24}O]^+$	Odd	$C_{13}H_{28}$ loss	Y (1.16)
201.16378	2.64	201.163777	$[C_{15}H_{21}]^+$	Even	$C_{14}H_{29}$ and H_2O losses	Y (11.26)
178.13521	2.35	178.135217	$[C_{12}H_{18}O]^+$	Odd		Y (1.14)
202.17161	2.26	202.171602	$[C_{15}H_{22}]^+$	Odd		Y (0.88)
359.33086	1.79	359.330842	$[C_{25}H_{43}O]^+$	Even		Y (2.56)
304.31245	1.17	304.312453	$[C_{22}H_{40}]^+$	Odd		Y (1.03)
387.36238	1.14	387.362143	$[C_{27}H_{47}O]^+$	Even		N
229.19510	0.78	229.195077	$[C_{17}H_{25}]^+$	Even		Y (2.69)

Table S.17: The main sterol standard CID fragments, and whether they were present (P) or absent (A) in the corresponding mid-Kawangware CID spectra. NIS = not in standard compound CID spectrum.

**CID Fragment**	**Kawangware sample peak isolations with CID**		
	**m/z 386.35**	**m/z 414.39**	**m/z 412.37**
	**'Cholesterol'**	**'β-sitosterol'**	**'Stigmasterol'**
CH_3 loss	P	P	P
H_2O loss	P	P	P
CH_3 and H_2O losses	P	P	P
C_3H_7O loss (Fig. S.13*(a)*)	P	P	A
C_5H_9O loss (Fig. S.13 *(b)*)	P	P	A
Side chain loss (Fig. S.13 *(e)*)	P	P	A (NIS)
Side chain and H_2O losses (Fig. S.13 *(f)*)	P	P	P
Side chain (plus H_2) loss (Fig. S.13 *(g)*)	A (NIS)	A (NIS)	P
Side chain (plus H_2) and H_2O losses (Fig. S.13 *(h)*)	A (NIS)	A (NIS)	P
Side chain plus C_3H_6 loss (Fig. S.14 *(i)*)	P	P	P
Side chain plus C_3H_6 and H_2O losses (Fig. S.14 *(j)*)	P	P	P
Side chain plus C_4H_8 loss (Fig. S.14 *(o)*)	A (NIS)	P	A
Side chain plus C_4H_8 and H_2O losses (Fig. S.14 *(p)*)	P	P	P

Table S.18. The main stanol standard CID fragments, and whether they were present (P) or absent (A) in the corresponding mid-Kawangware CID spectra. NIS = not in standard compound CID spectrum.

**CID Fragment**	**Kawangware sample peak isolations with CID**		
	**m/z 388.37**	**m/z 388.37**	**m/z 416.40**
	**'Coprostanol'**	**'Cholestanol'**	**'5α-sitostanol'**
CH_3 loss	P	P	P
H_2O loss	P	P	P
CH_3 and H_2O losses	P	P	P
$C_8H_{14}O$ loss (Fig. S.13 *(c)*)	P	P	P
$C_6H_{12}O$ loss (Fig. S.13 *(d)*)	P	P	P
Side chain plus C_3H_6 loss (Fig. S.14 *(k)*)	P	P	P
Side chain plus C_3H_5 loss (Fig. S.14 *(l)*)	P (NIS)	P	P
Side chain plus C_3H_6 and H_2O losses (Fig. S.14 *(m)*)	P	P	P
Side chain plus C_3H_5 and H_2O losses (Fig. S.14 *(n)*)	P (NIS)	P	P
Side chain plus C_4H_8 loss (Fig. S.14 *(q)*)	P (NIS)	P	P
Side chain plus C_4H_8 and H_2O losses (Fig. S.14 *(r)*)	P	P	P

Supplementary Figures

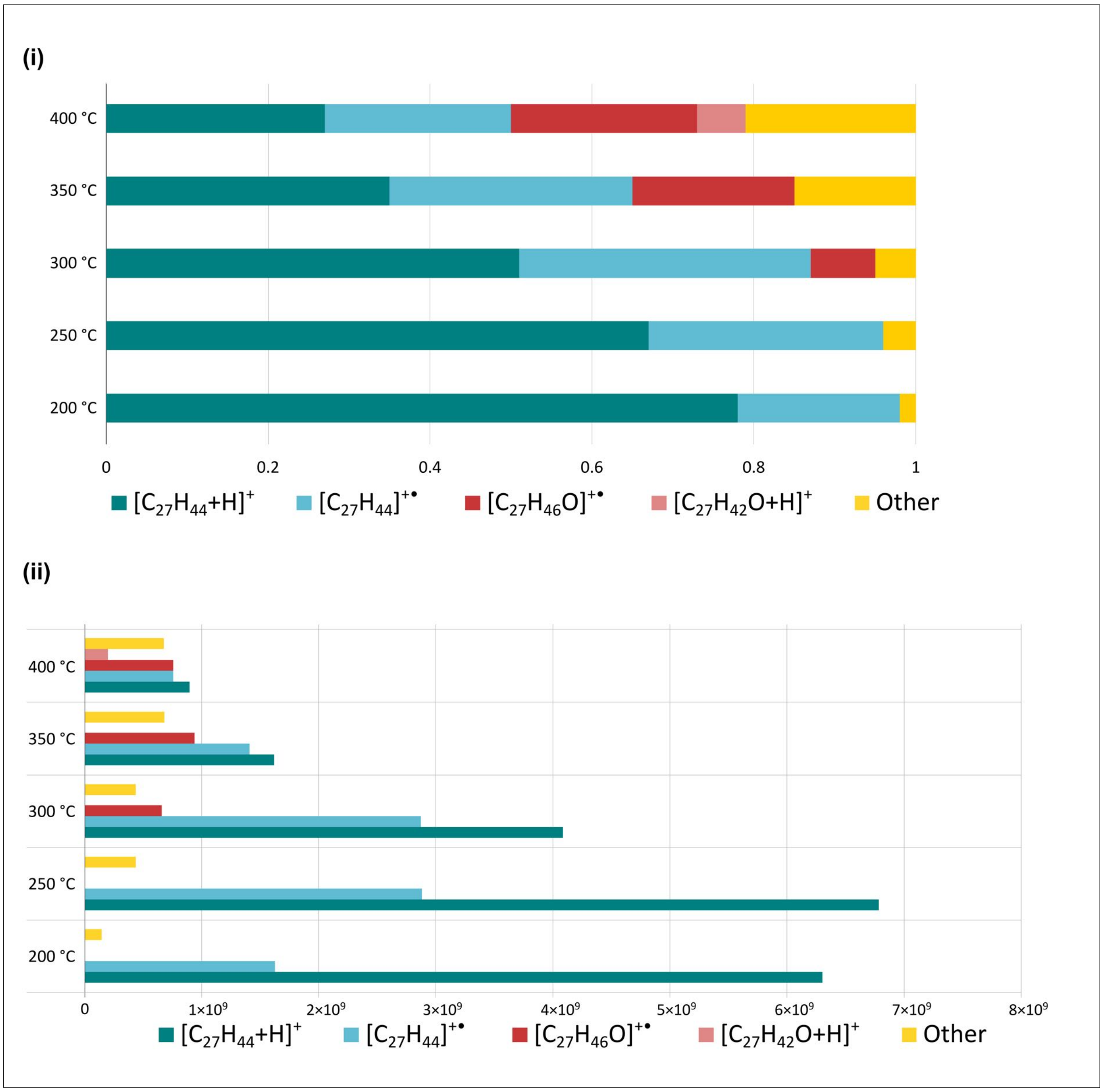

Figure S.1. Cholesterol ion species detected via broadband (+)APPI-FTICR MS, with the source vaporizer set to different temperatures. (i) Scaled signal contributions for the main ion species. (ii) Absolute signal intensity for the main ion species.

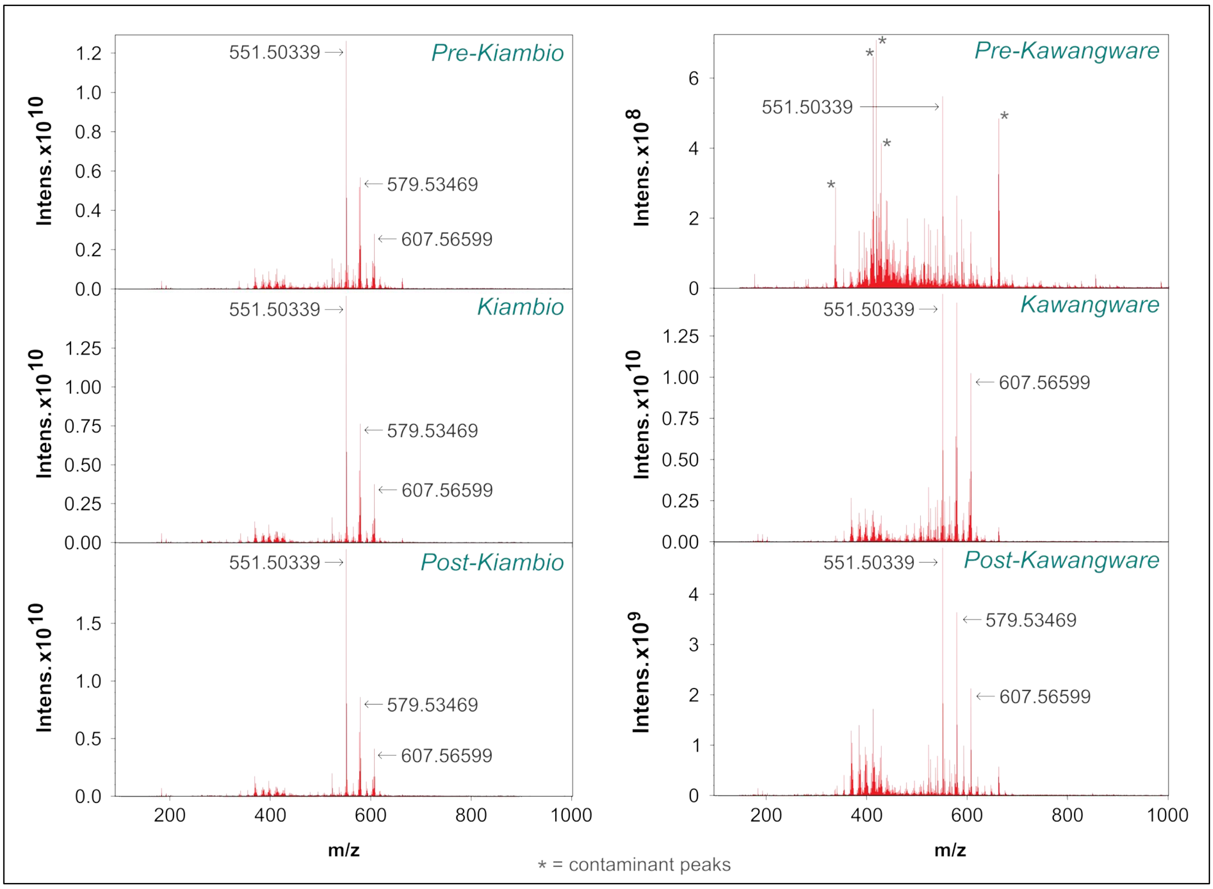

Figure S.2. Broadband APPI-FTICR MS overviews of the Nairobi sediments (magnitude mode).

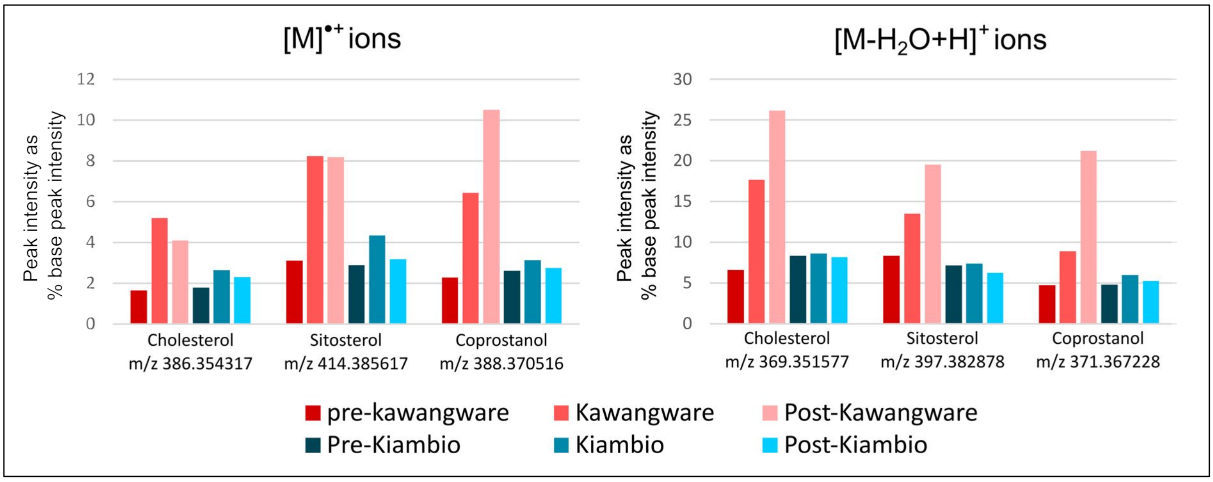

Figure S.3. Relative intensities of detected ions potentially representing the steroids cholesterol, β-sitosterol, and coprostanol.

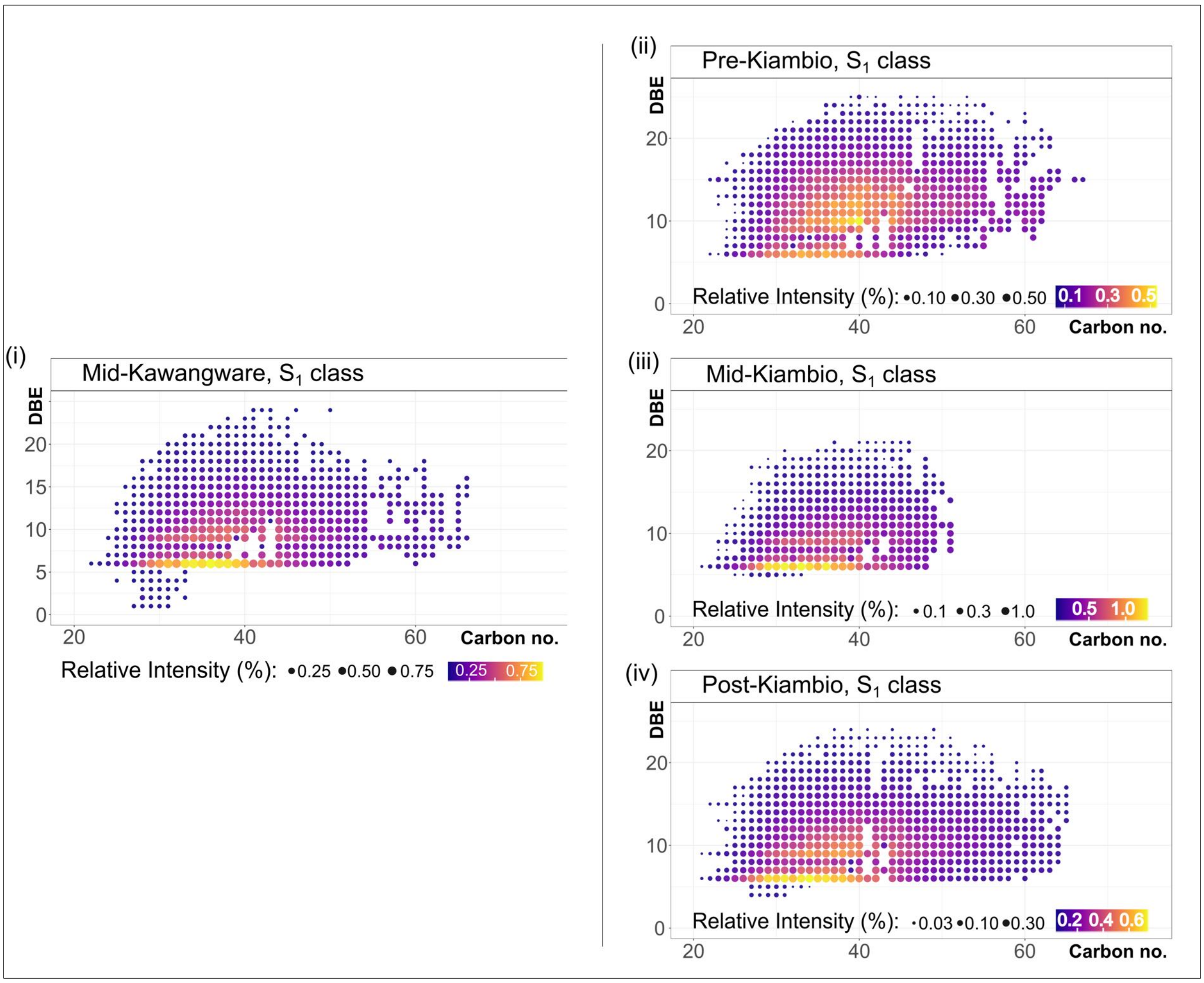

Figure S.4. S_1 class DBE versus carbon number plots (monoisotopic). (i) Kawangware, (ii)-(iv) Kiambio sites.

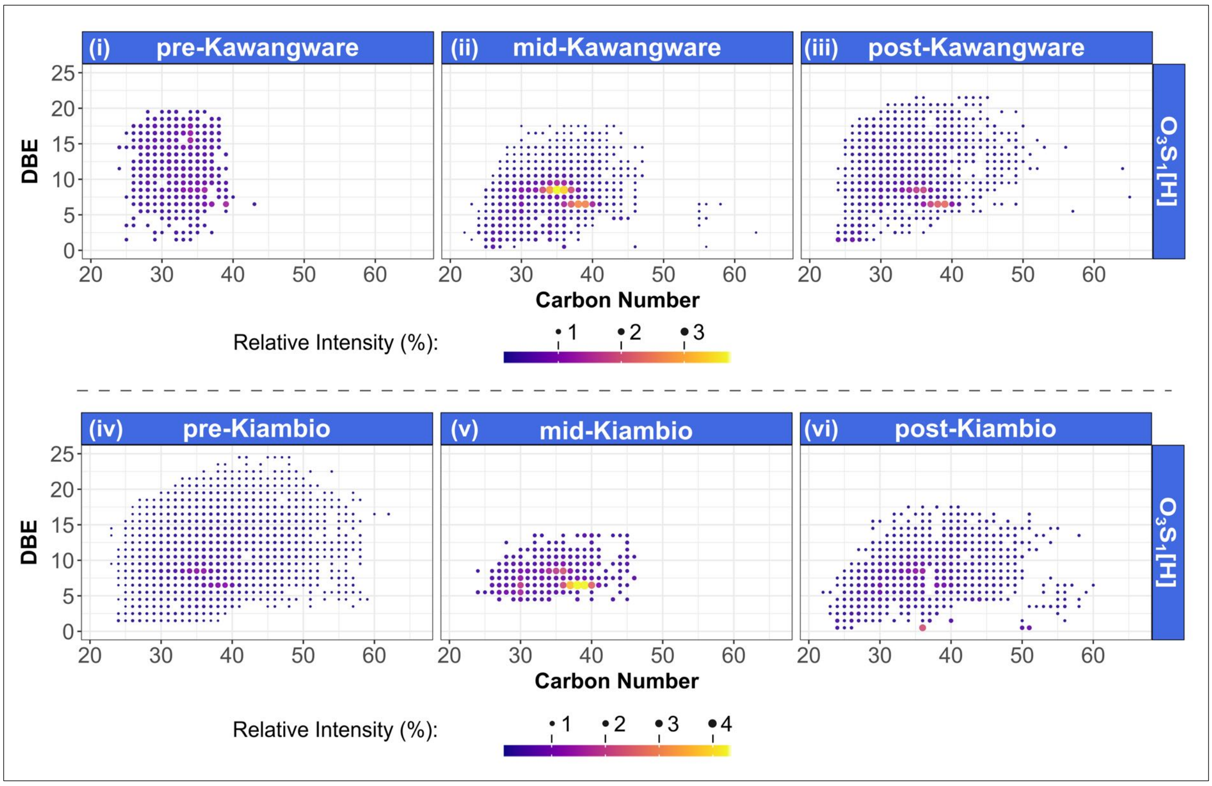

Figure S.5. $O_3S_1[H]$ class DBE versus carbon number plots (monoisotopic). (i)-(iii) Kawangware sites, (iv)-(vi) Kiambio sites.

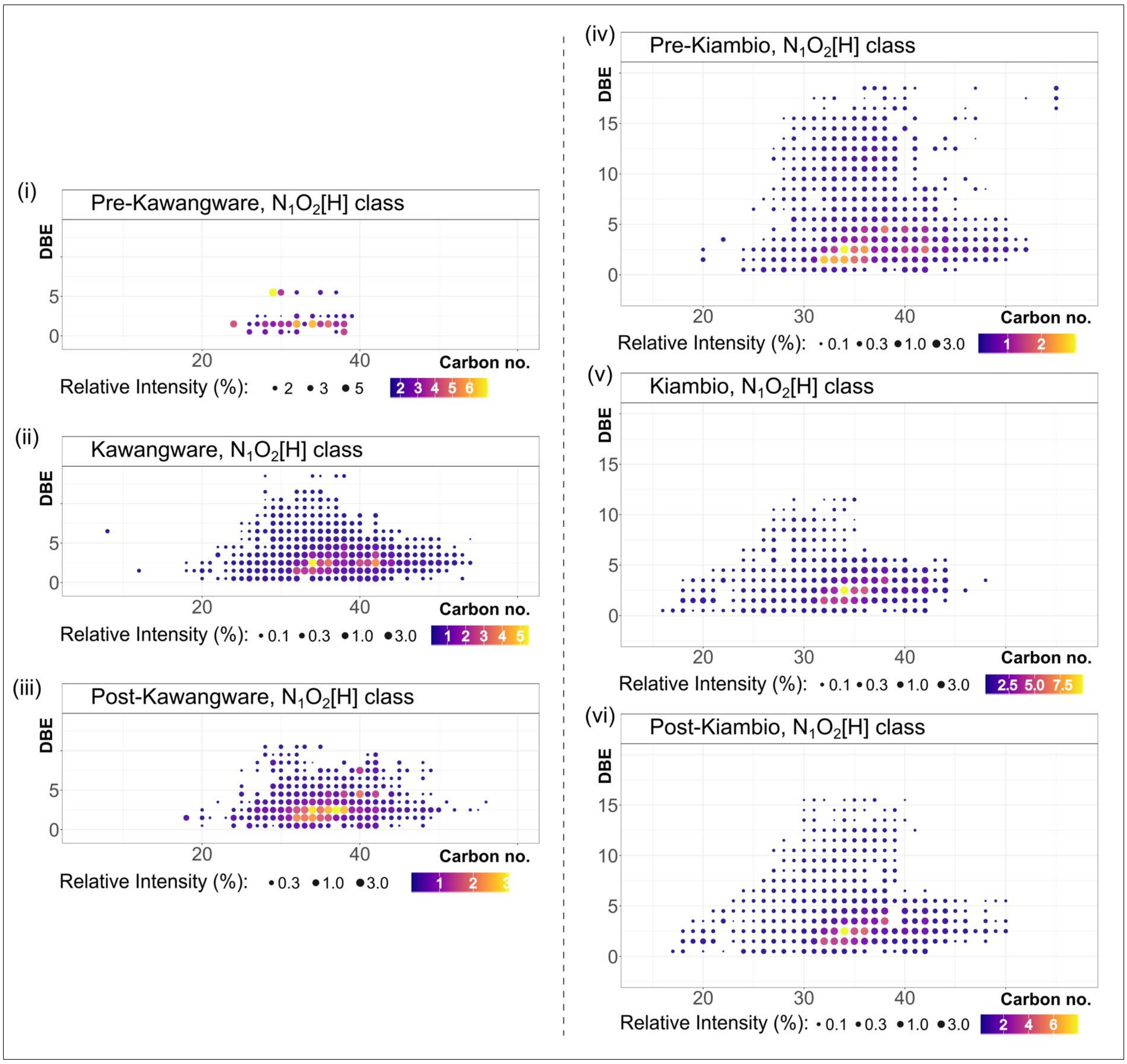

Figure S.6. $N_1O_2[H]$ class DBE versus carbon number plots (monoisotopic). (i)-(iii) Kawangware sites, (iv)-(vi) Kiambio sites.

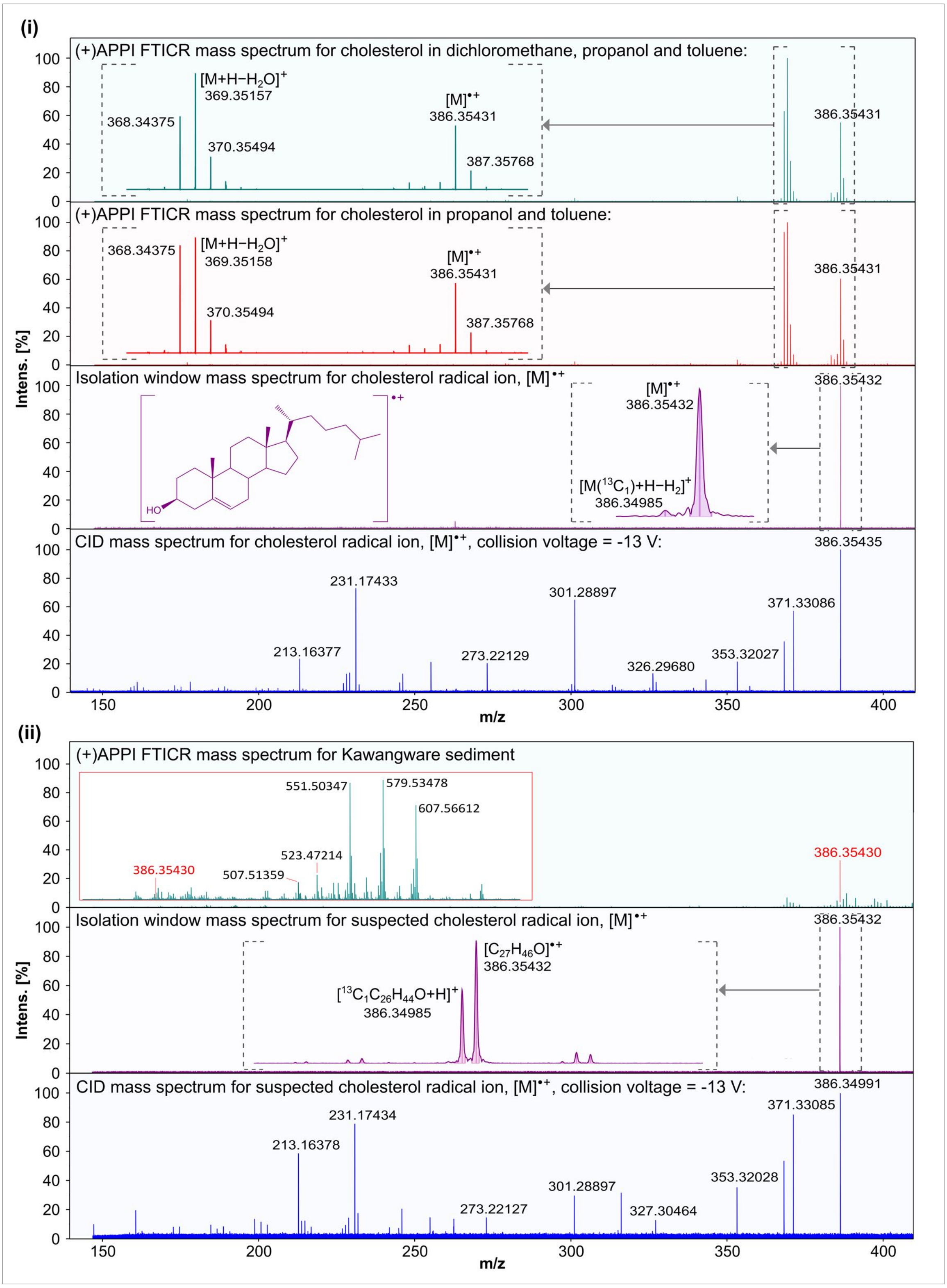

Figure S.7. APPI FTICR broadband, isolation, and CID spectra for the cholesterol standard (i) and Kawangware sample (ii). N.B. m/z values reflect independent recalibrations.

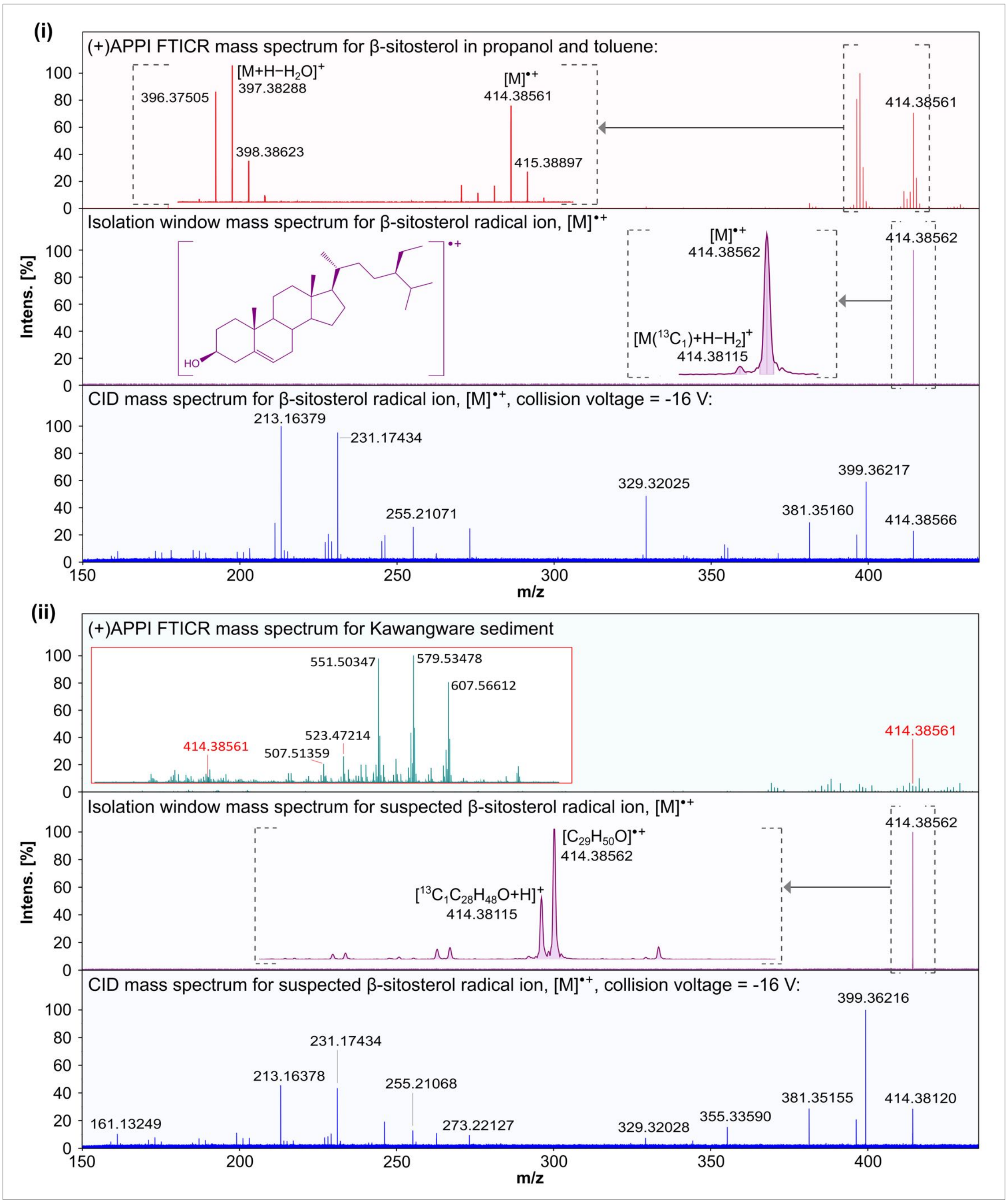

Figure S.8. APPI FTICR broadband, isolation, and CID spectra for the β-sitosterol standard (i) and Kawangware sample (ii). N.B. m/z values reflect independent recalibrations.

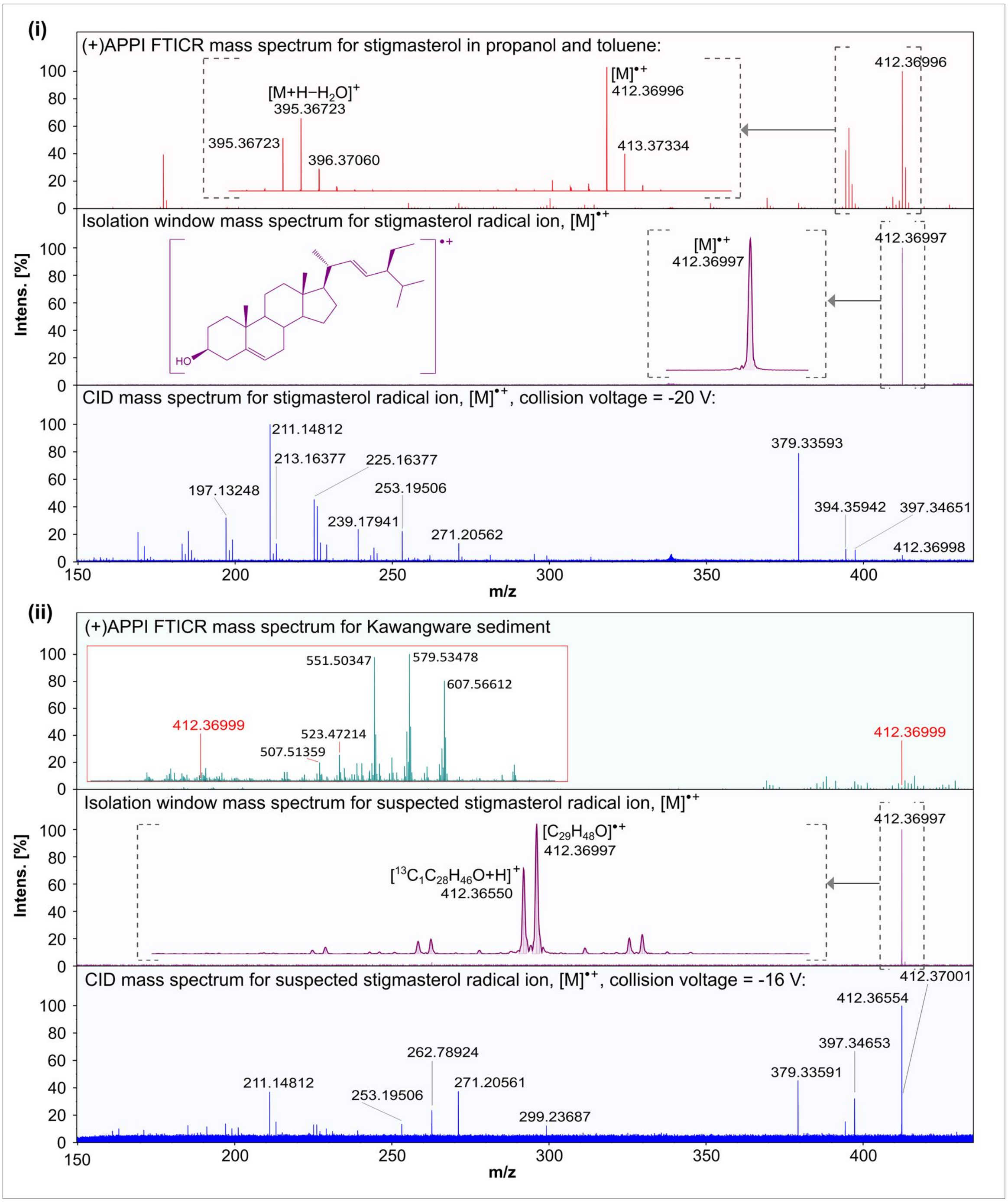

Figure S.9. APPI FTICR broadband, isolation, and CID spectra for the stigmasterol standard (i) and Kawangware sample (ii). N.B. m/z values reflect independent recalibrations.

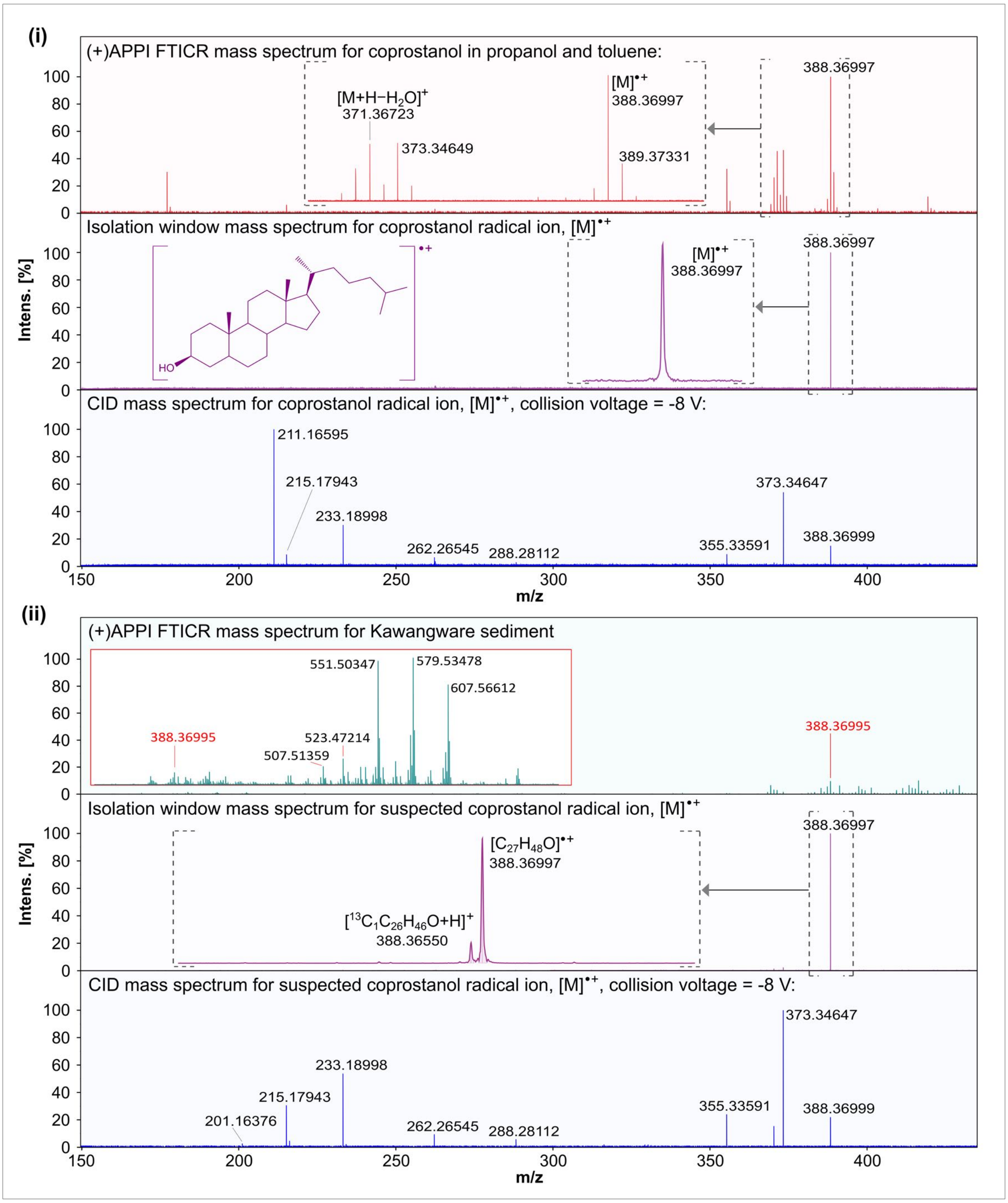

Figure S.10. APPI FTICR broadband, isolation, and CID spectra for the coprostanol standard (i) and Kawangware sample (ii). N.B. m/z values reflect independent recalibrations.

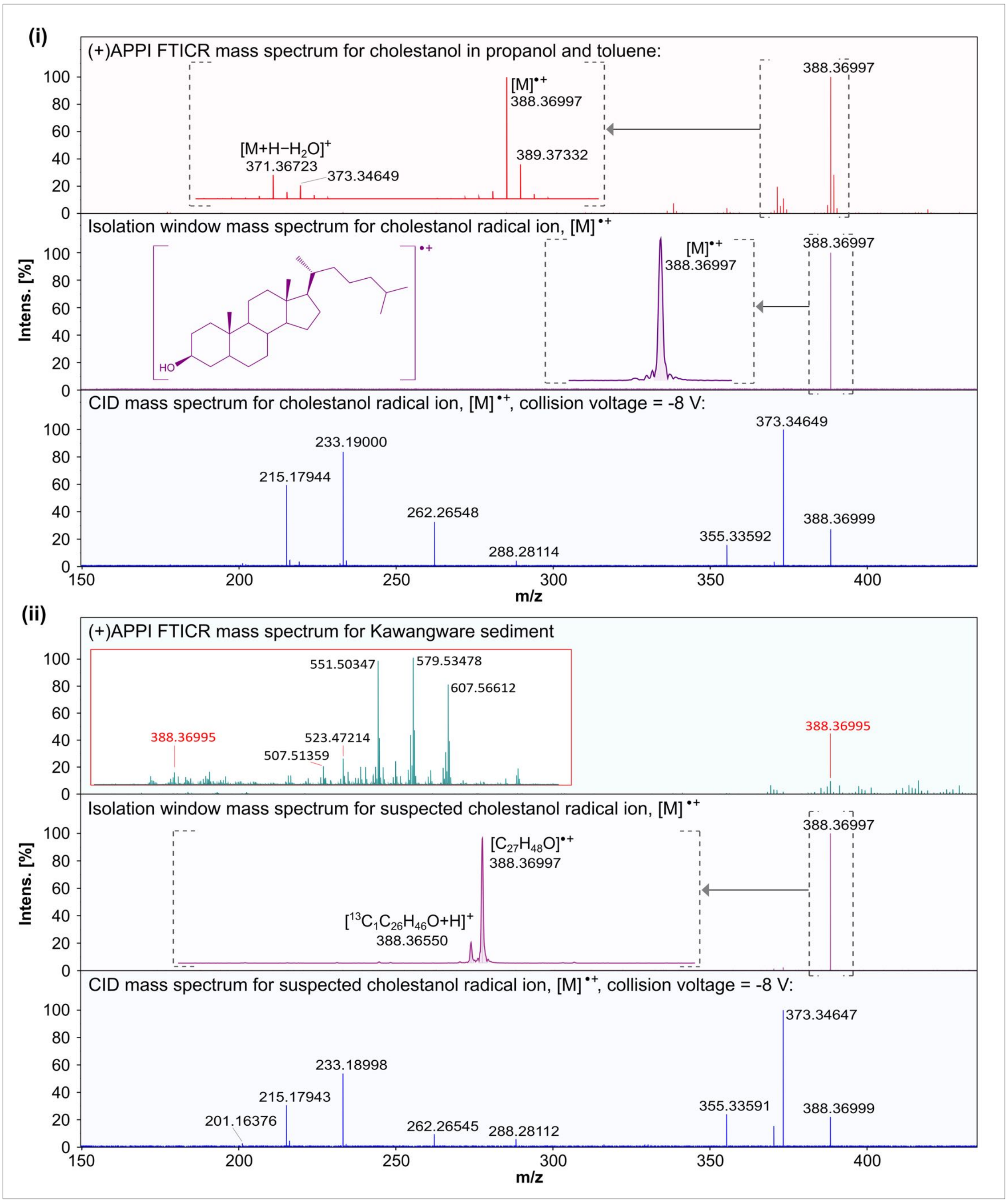

Figure S.11. APPI FTICR broadband, isolation, and CID spectra for the cholestanol standard (i) and Kawangware sample (ii). N.B. m/z values reflect independent recalibrations.

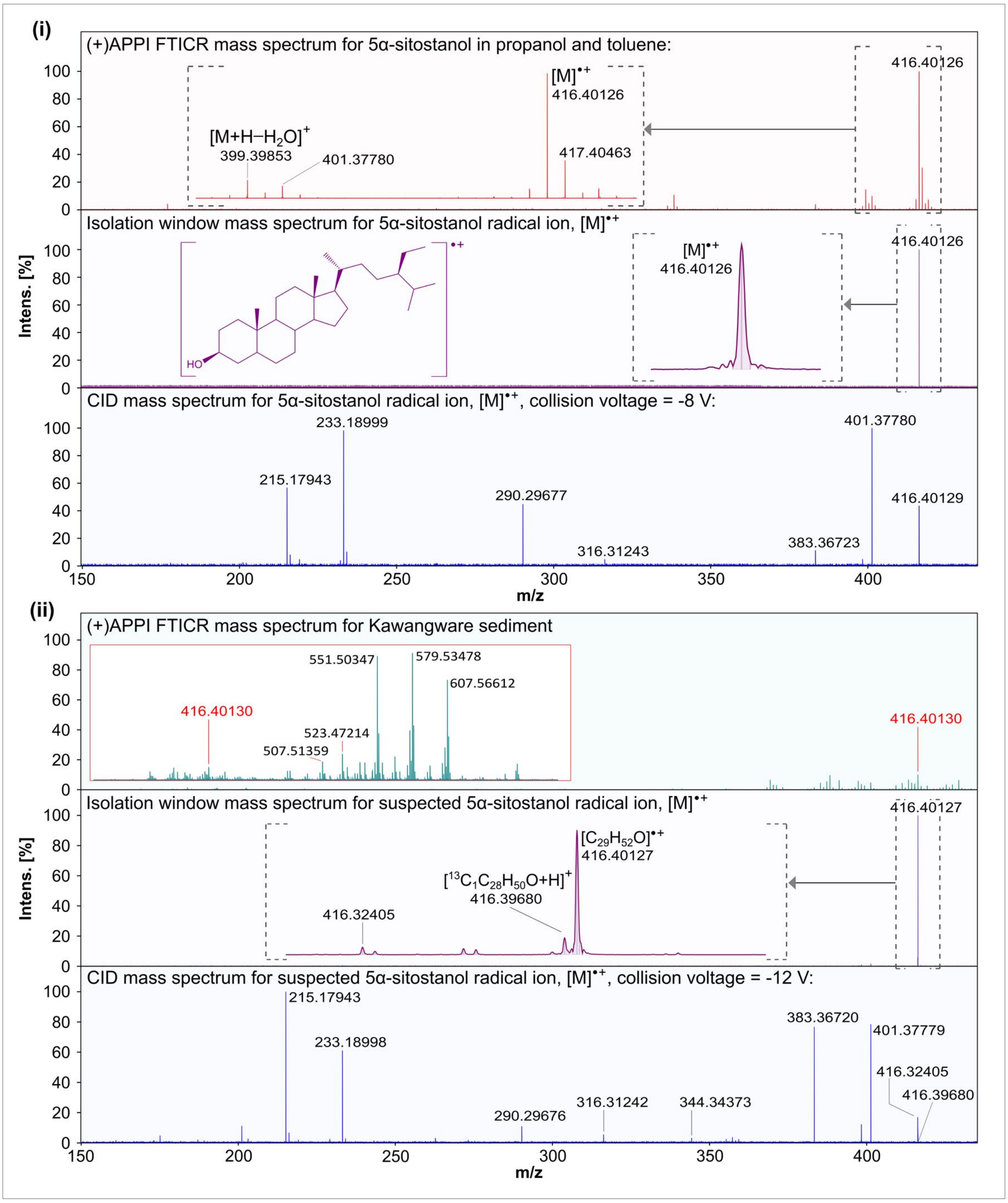

Figure S.12. APPI FTICR broadband, isolation, and CID spectra for the 5α-sitostanol standard (i) and Kawangware sample (ii). N.B. m/z values reflect independent recalibrations.

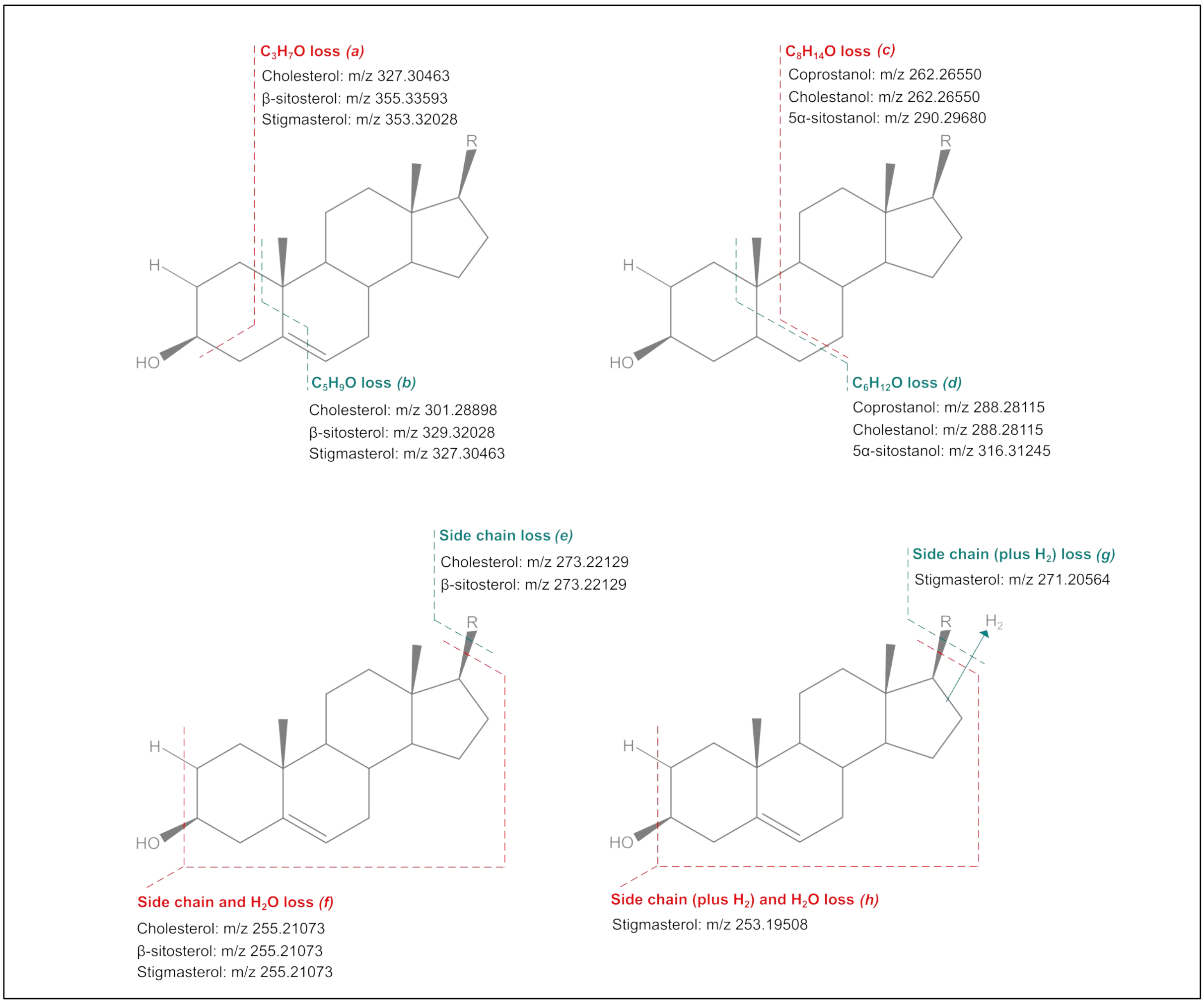

Figure S.13. A selection of proposed sterol and stanol fragmentations as measured for standards by APPI-FTICR MS with CID. Fragmentation (a) proposed by Nakakuni et al.[3]. Fragmentation (b) and (e) proposed by van Agthoven et al.[1] and West and Reid[2]. Fragmentation (f) proposed by Moreau et al.[4]. N.B. side chain cleavages as per (e) was not observed for stanols.

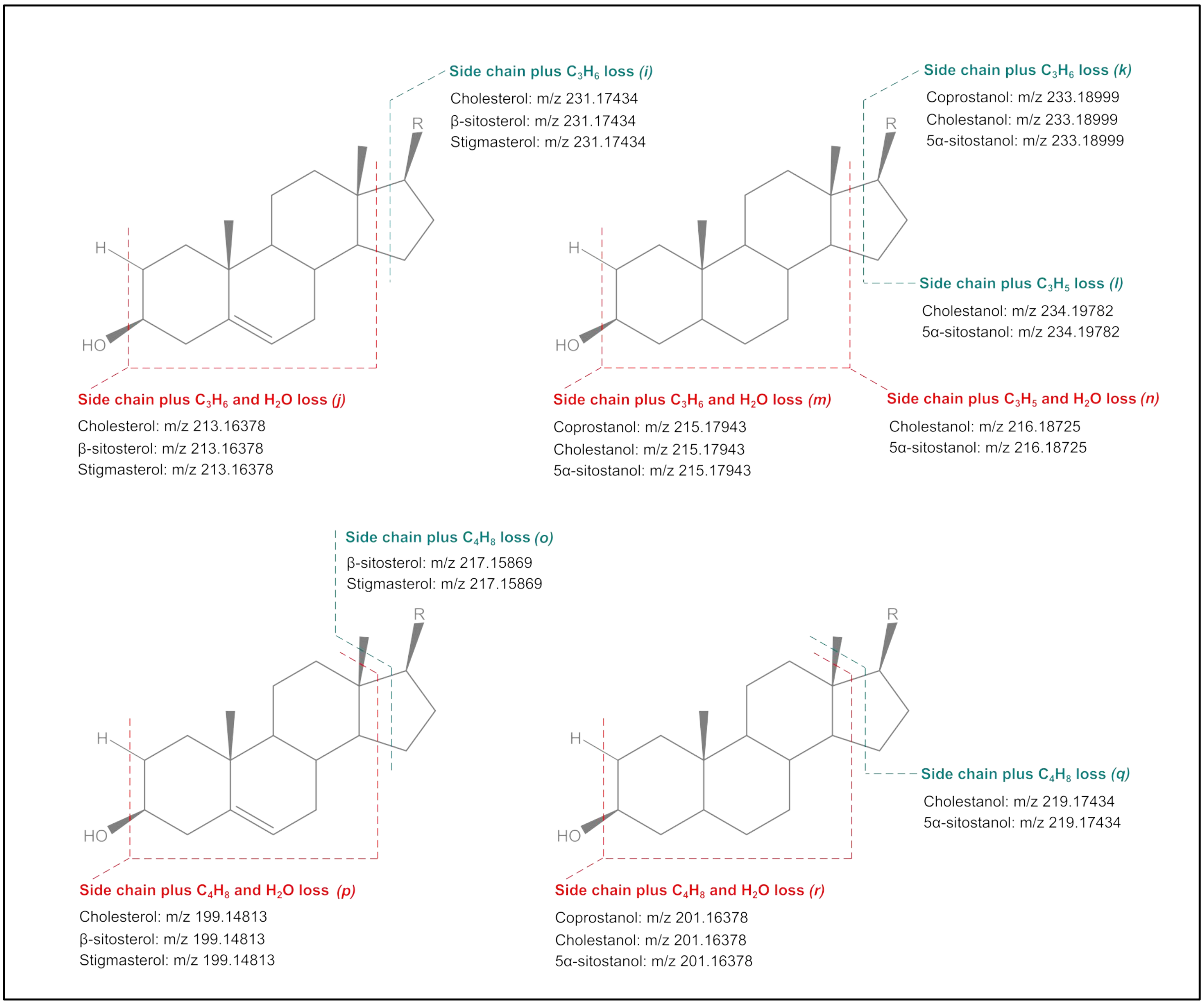

Figure S.14. A selection of proposed sterol and stanol fragmentations as measured for standards by APPI-FTICR MS with CID. Fragmentation (i) proposed by van Agthoven et al.[1] and West and Reid[2]. Fragmentation (l) proposed by Nakakuni et al.[3]. N.B. a m/z 201.16278 peak was also observed for β-sitosterol and stigmasterol, and a m/z 215.17943 peak was detected for β-sitosterol.

References

1. van Agthoven, M.; Barrow, M.; Chiron, L.; Coutouly, M.; Kilgour, D.; Wootton, C.; Wei, J.; Soulby, A.; Delsuc, M.; Rolando, C.; et al. Differentiating Fragmentation Pathways of Cholesterol by Two-Dimensional Fourier Transform Ion Cyclotron Resonance Mass Spectrometry. *J. Am. Soc. Mass Spectrom.* **2015**, *26* (12), 2105-2114. DOI: 10.1007/s13361-015-1226-7

2. West, H.; Reid, G. Hybrid 213 nm Photodissociation of Cationized Sterol Lipid Ions Yield $[M]^{+\bullet}$ Radical Products for Improved Structural Characterization Using Multistage Tandem Mass Spectrometry. *Anal. Chim. Acta* **2021**, *1141*, 100-109. DOI: 10.1016/j.aca.2020.10.013

3. Nakakuni, M.; Yamasaki, Y.; Yoshitake, N.; Takehara, K.; Yamamoto, S. Methyl Ether-Derivatized Sterols and Coprostanol Produced via Thermochemolysis Using

Tetramethylammonium Hydroxide (TMAH). *Molecules* **2019**, *24* (22). DOI: 10.3390/molecules24224040

4. Moreau, R.; Whitaker, B.; Hicks, K. Phytosterols, Phytostanols, and their Conjugates in Foods: Structural Diversity, Quantitative Analysis, and Health-Promoting Uses. *Prog. Lipid Res.* **2002**, *41* (6), 457-500. DOI: 10.1016/S0163-7827(02)00006-1
